# Glycoprotein G enables HSV-2 neuroinvasion and provides protection as a glycosylated vaccine antigen

**DOI:** 10.1371/journal.ppat.1014339

**Published:** 2026-07-09

**Authors:** Ebba Könighofer, Carolina Gustafsson, Lindvi Gudmundsdotter, Ekaterina Mirgorodskaya, Jonas Nilsson, Maria Ekblad, Beata Adamiak, Eva Jennische, Stefan Lange, Edward Trybala, Staffan Görander, Tomas Bergström, Jan-Åke Liljeqvist, Rickard Nordén

**Affiliations:** 1 Department of Infectious Diseases, Institute of Biomedicine, Sahlgrenska Academy, University of Gothenburg, Gothenburg, Sweden; 2 Department of Clinical microbiology, Sahlgrenska University Hospital, Västra Götalandsregionen, Sweden; 3 Simplexia AB, Gothenburg, Sweden; 4 Proteomics Core Facility, Sahlgrenska Academy, University of Gothenburg, Gothenburg, Sweden; 5 Department of Medical Biochemistry and Cell Biology, Institute of Biomedicine, Sahlgrenska Academy, University of Gothenburg, Gothenburg, Sweden; University of Illinois at Chicago, UNITED STATES OF AMERICA

## Abstract

The role of glycoprotein G (gG-2) of herpes simplex virus type 2 (HSV-2) in viral pathogenesis remains poorly understood. gG-2 is cleaved into a secreted form (sgG-2) and a membrane-associated form (mgG-2), but the in vivo function of mgG-2 and the contribution of its glycosylation to immune responses have not been defined. Here, we provide a comprehensive characterization of the N- and O-linked glycosylation profile of mgG-2 and investigate its functional relevance for viral spread and vaccine-induced immunity. Using a mouse genital infection model, we show that an mgG-2-deficient HSV-2 mutant replicates in vaginal epithelial cells but is severely impaired in dissemination to dorsal root ganglia and the central nervous system, identifying mgG-2 as a key determinant of neuronal spread in vivo. In parallel, immunization with recombinant mgG-2 elicited strong humoral and Th1-polarized CD4 + T-cell responses and conferred protection against genital HSV-2 challenge. Importantly, glycosylation of mgG-2 was required for optimal immunogenicity and protection, as deglycosylated variants induced reduced CD4 + T-cell responses and allowed increased viral spread to neuronal tissues. Mechanistically, our findings suggest that glycosylation of mgG-2 modulates antigen recognition and shapes adaptive immune responses that limit viral dissemination after vaccination. Together, these results demonstrate that mgG-2 plays a critical role in HSV-2 pathogenesis and provide a strong rationale for targeting glycosylated mgG-2 in the development of both prophylactic and therapeutic vaccines against HSV-2.

## Author summary

Herpes simplex virus type 2 infects hundreds of millions of people worldwide and establishes a lifelong infection. Despite decades of research, there is still no approved vaccine against this virus. A better understanding of how it spreads in the body and which viral components can trigger protective immunity is therefore important for future vaccine development. In this study, we identified a previously unrecognized role for the viral protein membrane-associated glycoprotein G. We found that this protein contributes to the virus’s ability to invade the nervous system, providing new insight into how infection becomes established and how disease develops. At the same time, we showed that a glycosylated form of the same protein can induce protective immune responses when used as a vaccine antigen. Our findings improve our understanding of how this virus causes infection and highlight a promising new target for vaccine development. By identifying a viral protein that is involved both in disease processes and in the induction of protective immunity, our work may help guide future efforts to develop vaccines and other strategies to reduce the global burden of herpes simplex virus type 2 infection.

## Introduction

Human herpes simplex virus type 2 (HSV-2) is classified into the *Alphaherpesvirinae* subfamily due to its capacity to establish latency in sensory neurons. HSV-2 infects the genital mucosa and establishes a lifelong latent infection in sensory neurons of dorsal root ganglia (DRG) located in the lumbosacral region of the spine. After reactivation in DRG, the virus is antegradely transported to the periphery, causing genital lesions or asymptomatic shedding of the virus. HSV-2 is also a significant cause of infections within the central nervous system (CNS), arising either during the primary infection or by recurrent reactivation of latent virus [1, 2]. In 2016, it was estimated that over 500 million people aged 15–49 years were infected worldwide [3]. Two prophylactic vaccine candidates, based on HSV-2 envelope glycoprotein gB-2 and/or gD-2, have reached phase III clinical trials but failed to prevent HSV-2 infection, reviewed in [4]. The limited outcomes have been attributed, in part, to the inability of these candidates to elicit sufficiently broad and durable immune responses, as well as to viral immune evasion mechanisms that may impair Fc-mediated effector functions, including antibody-dependent cellular cytotoxicity (ADCC). These challenges highlight the need to explore alternative viral antigens that may induce complementary or more effective protective immune responses.

HSV-2 encodes 12 envelope glycoproteins responsible for various functions, including viral entry, egress, cell-to-cell spread, and neuronal transport. Like several other envelope glycoproteins, such as gC, gI and gE [5, 6, 7, 8], the glycoprotein G of HSV-2 (gG-2) is dispensable for virus propagation in cell cultures [9]. During protein maturation, gG-2 is post-translationally cleaved into a secreted amino-terminal protein (sgG-2) and a carboxy-terminal high mannose-containing intermediate that is further processed by N- and O-linked glycosylation, to constitute the cell-membrane anchored mature gG-2 (mgG-2) [10,11,12,13,14,15–17]. While the exact glycosylation structures have not been established, the amino-terminal half of mgG-2 is predicted to be a mucin-like region based on the high abundance of the amino acid residues proline, serine, threonine and alanine. Such mucin-like domains are typically heavily O-glycosylated. Extensive glycosylation in this region may contribute to immune modulation by altering antigen processing, or modulating the antigenicity of specific sites, thereby potentially shaping both humoral and cellular immune responses [18,19,20,21,22]. Previous studies have begun to characterize the glycosylation of gG-2, demonstrating that both N- and O-linked glycans are present [23, 12, 24]. However, a comprehensive mapping of glycosylation sites and a functional assessment of how glycosylation influences immune responses to mgG-2 remain limited.

Both sgG-2 and mgG-2 stimulate HSV-2 type-specific B- and T-cell responses [25, 26, 27, 28, 29], and mgG-2 is widely used as an antigen for type-discriminating serology. Functional studies also show that a sgG-2 peptide functions as a chemoattractant for both monocytes and neutrophils [30], and enhances chemotaxis and chemokine function [31]. Furthermore, sgG-2 also modifies nerve growth factor signalling to attract free nerve endings at the site of infection, possibly contributing to novel immune escape mechanisms utilized by HSV-2 [32]. While the functional role of mgG-2 in human genital infection and subsequent neuronal spread remains unclear, data demonstrate its potential as a vaccine candidate in a mouse genital challenge model, suggesting that the immune response against mgG-2 can inactivate important functions of the protein in primary HSV-2 infection [33]. An mgG-2 negative HSV-2 strain was shown to spread mostly from cell to cell and had an impaired capacity to release HSV-2 virions into the extracellular medium of the cell culture. In addition, the trapped virions at infected cell membranes were fully virulent and could be released from the cells with sulphated oligosaccharides such as heparin [34–36].

**Fig 1:**
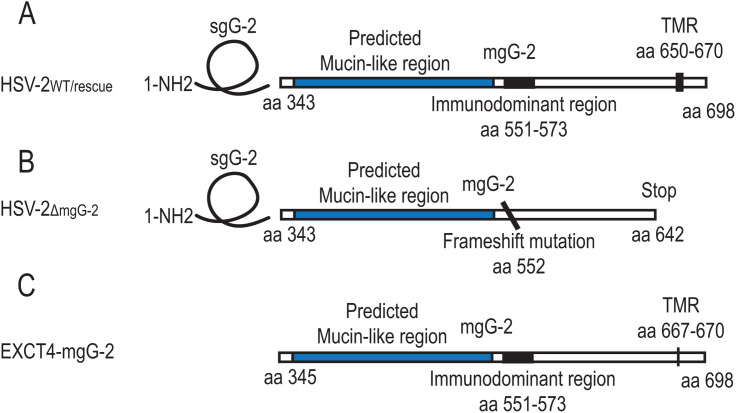
Schematic illustration of the gG-2 protein of HSV-2 and its variants used in the current study. **(A)** The wild type gG-2 protein of HSV-2_WT_ is cleaved generating a secreted portion (sgG-2) and a cell membrane-anchored portion (mgG-2). HSV-2_rescue_ was generated using a marker transfer assay and restores the HSV-2_WT_ genotype. **(B)** The recombinantly produced EXCT4-mgG-2 contains the extracellular region, four amino acids of TMR, and the entire intracellular region. **(C)** The HSV-2_ΔmgG-2_ mutant harbours a frame shift mutation within nucleotides coding for amino acid 552 generating a premature stop at amino acid 642. The immunodominant region for antibodies is marked including the amino acids 551-573. The predicted mucin-like region is indicated. The trans-membrane region is denoted TMR.

In this work, the role of mgG-2 during *in vivo* infection and the potential use of mgG-2 as a vaccine antigen against HSV-2 infection, was explored. To evaluate the vaccine potential of mgG-2, we employed the EXCT4 platform, a recombinant protein-based vaccine system designed to enhance antigen stability and immunogenicity [33, 37]. Additionally, we aimed to delineate the N- and O-linked glycosylation patterns of a recombinant mgG-2 subunit vaccine candidate and evaluate how the glycosylation profile influence protective immune responses against HSV-2 challenge. To dissect the functional role of mgG-2 in vivo and to assess its role in neuronal spread, we used HSV-2 wild type strain 333 (HSV-2_WT_, Fig 1A), an mgG-2 negative mutant (HSV-2_ΔmgG-2,_ (Fig 1B), and a rescued HSV-2 strain in which the mgG-2 gene was reintroduced (HSV-2_rescue_) in a genital infection model across two mouse strains.

## Results

### Recombinant EXCT4-mgG-2 is decorated with sialylated N- and O-linked glycans

**Fig 2:**
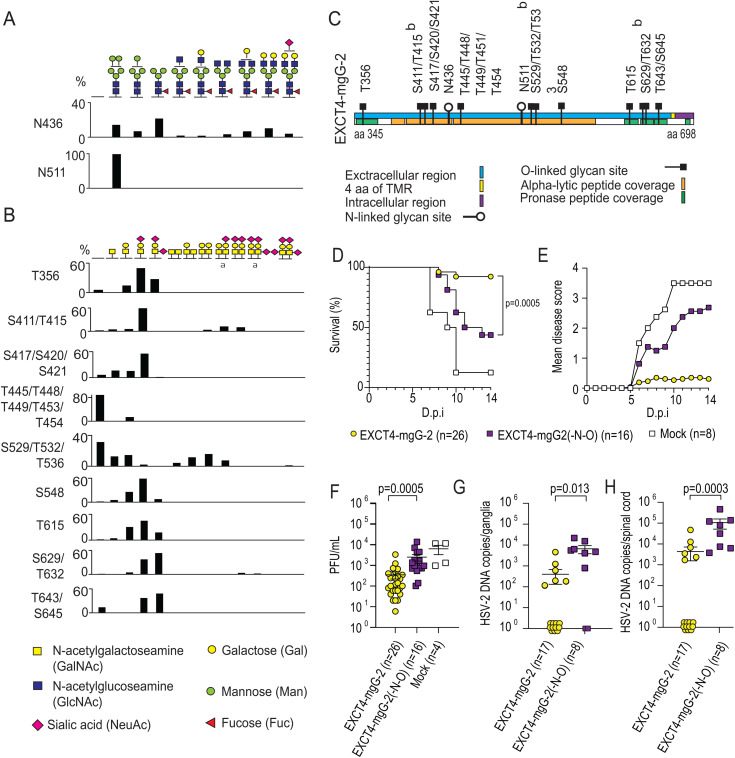
Immunization with EXCT4-mgG-2 containing multiple N- and O-linked glycan structures, facilitates protection against neuronal spread of HSV-2 in mice. **(A)** Distribution between different glycoforms at each identified glycosite for N-linked glycans. **(B)** Distribution between different glycoforms at each identified glycosite for O-linked glycans. The exact glycosite could not be determined for all O-linked glycans, all possible sites for the respective glycan are stated and indicated with “/”. In the cases where more than one O-linked glycan was observed on the same peptide, two separate glycoforms are present on the same peptide in the schematic drawing. Black bars indicate the percentage of the identified glycoforms on each respective site. Monosaccharide symbols are represented according to the Symbol Nomenclature for Glycans (SNFG) system [38]. **(C)** Schematic drawing of EXCT4-mgG-2, including peptide coverage using alphalytic and pronase-treatment, and glycosites for N-linked (black) and O-linked (white) glycosylation. All detected O-linked glycans, regardless of the frequency, are indicated. **(D-E)** C57BL/6 mice were intramuscularly immunized with EXCT4-mgG-2 and EXCT4-mgG-2(−N − O) and genitally challenged with 25 x LD_50_ of HSV-2_WT_. The survival rate and disease score were assessed until 15 d.p.i. **(F)** The number of HSV-2 plaque forming units (PFU) in vaginal washings 2 d.p.i. **(G)** HSV-2 DNA copies per ganglia at 14 d.p.i. **(H)** HSV-2 DNA copies per spinal cord at 2 d.p.i. The detection limit for plaque forming units in vaginal washings was 20 PFU/mL and 40 or 160 HSV-2 DNA copies perganglia (3 ganglion) or whole spinal cord respectively. Statistical analysis was performed with the pairwise log-rank (Mantel Cox) test **(D)**, and Kruskal-Wallis test **(F-H)**. D.p.i. - days post infection. Values are expressed as means ± SEM. ^a^ The sialic acid has two possible sites, but the exact position could not be determined. ^b^ More than one O-linked glycan was observed on the same peptide.

First, we produced a recombinant truncated mgG-2 (EXCT4-mgG-2) in Chinese Hamster Ovary (CHO-K1) cells (Fig 1C). Using liquid chromatography tandem mass spectrometry (LC-MS/MS) analysis of protease-cleaved glycopeptides, we dissected the glycan profile of the recombinant protein (Fig 2A-2C). Protease cleavage of the EXCT4-mgG-2 prior to LC-MS/MS was performed using alphalytic and pronase. Representative peptides, covering all N-linked consensus sites and 63 out of 70 (90%) possible sites for O-linked glycosylation, were identified and curated for glycan composition and abundance (S1A Fig and S1 Table). Peptide stretches where the fragment spectra evaluation could not confirm the identity of the peptides were excluded from the analysis.

EXCT4-mgG-2 contains three sites with consensus sequence Asn-x-Ser/Thr (x = any amino acid, except Pro), which allow for N-linked glycosylation, of these, two (N436 and N511) were identified to be glycosylated (S1B Fig and S2 Table). Site N436 was found to be glycosylated on all identified peptides, primarily by complex type glycans (39.8%) with the most processed structure identified consisted of GlcNAc4Man3Gal2Fuc1NeuAc1, while the oligomannose and paucimannose structures at site N436 were of similar levels (29.8% and 30.4% respectively) (Fig 2A). Site N511 was found to be exclusively decorated with high mannose (Man5GlcNAc2) (Fig 2A). Of the 70 possible sites (Ser/Thr) for O-linked glycosylation, 12 were found to carry glycan structures (Fig 2B). Overall, the most prevalent glycoform was the T-antigen (GalNAc1Gal1), which was found both non-, mono- and di-sialylated. Of note, two of the peptides carrying glycoforms of the N-linked glycan of N436, were also identified as simultaneously carrying an O-linked glycan structure. However, this O-linked structure could not be independently validated when the glycan search was restricted to an O-glycan library alone.

### EXCT4-mgG-2 protection against HSV-2 infection is glycosylation dependent

Next, enzymatic treatment protocols were used to remove specific glycan structures from EXCT4-mgG-2, generating five alternative antigens 1: N- and O-glycosylated (EXCT4-mgG-2), 2: lacking both N- and O-linked glycans (EXCT4-mgG-2(−N − O)), 3: lacking N-linked glycans (EXCT4-mgG-2(-N)), 4: lacking O-linked glycans (EXCT4-mgG-2(-O)), or 5: lacking sialic acids (EXCT4-mgG-2(-SA)). Glycan trimming was confirmed by western blot and lectin binding-assay (S2 Fig). Next, C57BL/6 mice were immunized intramuscularly three times with respective antigen, followed by genital challenge with 25 x lethal dose 50% (LD_50_) of HSV-2_WT_ and thereafter the survival, disease score, viral replication and spread were monitored. Immunization with EXCT4-mgG-2 conferred protection, showing 93.7% survival 14 days post infection (d.p.i.) (n = 26), while immunization with EXCT4-mgG-2(−N − O) resulted in a survival of 43.8% (p = 0.0005, n = 16) (Fig 2D). The mean disease score for mice immunized with EXCT4-mgG-2 was 0.3 at 14 d.p.i., as compared to a mean disease score of 2.7 for the mice immunized with EXCT4-mgG-2(−N − O) (Fig 2E). The viral load was higher in vaginal washes from mice that were immunized with the deglycosylated antigen EXCT4-mgG-2(−N − O) compared to mice that received EXCT4-mgG-2 (p = 0.0005) (Fig 2F). Mice immunized with EXCT4-mgG-2(−N − O) presented with higher levels of HSV-2 DNA in DRG than mice immunized with EXCT4-mgG-2, 48 hours post infection (h.p.i.) (p = 0.013) (Fig 2G). Also, the HSV-2 DNA copy numbers in the spinal cord were significantly higher in mice immunized with EXCT4-mgG-2(−N − O) (p = 0.0003) (Fig 2H), indicating poorer protection against viral spread in neuronal tissue. In contrast, immunization with EXCT4-mgG2(-N) (n = 8), EXCT4-mgG-2(-O) (n = 8) or EXCT4-mgG-2(-SA) (n = 8), all conferred protection against HSV-2_WT_ and the mice showed similar disease score as mice immunized with EXCT4-mgG-2 (S3A-S3B Fig). In addition, no significant impairment in the protective effect on viral spread in neuronal tissue was observed compared to EXCT4-mgG-2 (S3C-S3D Fig). In conclusion, negative effect on the immune response occurs when both the N- and O-linked glycans are removed from the antigen.

### EXCT4-mgG-2 generates a glycosylation dependent adaptive immune response

**Fig 3:**
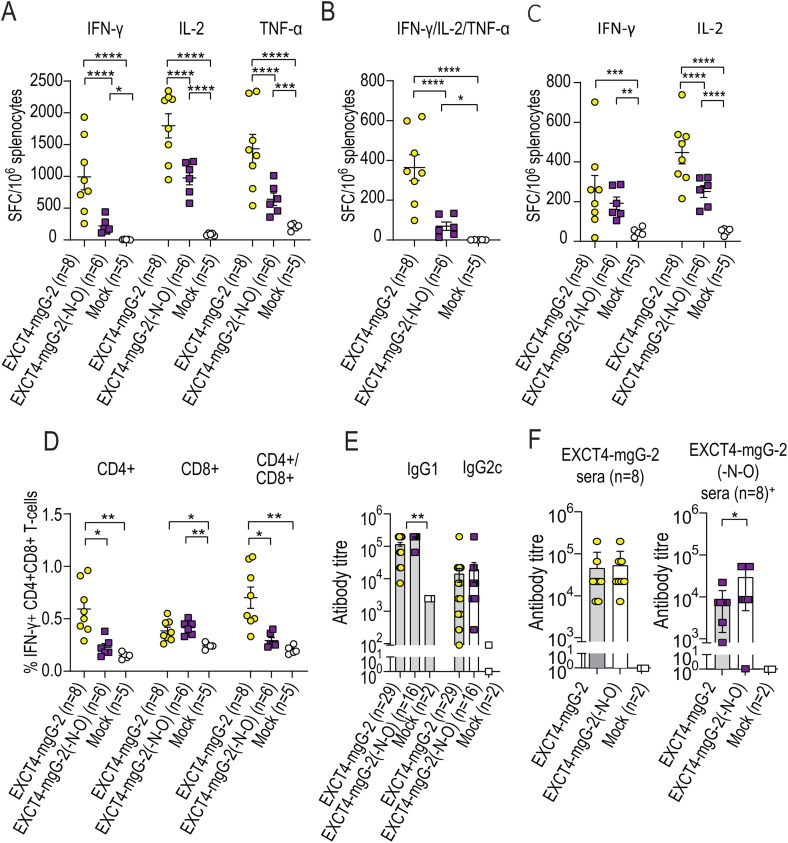
Immunization with EXCT4-mgG-2 induces Th1 polarization, a response which is attenuated in the absence of glycosylation. **(A)** Flourospot analysis of splenocytes harvested after immunization and stimulated with a pool of overlapping peptides (15-mers) covering the entire EXCT4-mgG-2 sequence, showing INF-γ, IL-2 and TNF-α production. **(B)** Fluorospot analysis showing the combined expression of INF-γ, IL-2 and TNF-α in splenocytes stimulated with the 15-mer peptide pool **(C)** Fluorospot analysis showing INF-γ and IL-2 expression in splenocytes stimulated with the N- and O-glycosylated EXCT4-mgG-2 recombinant protein **(D)** Percentage of INF-γ positive CD4+ and CD8 + T cells from splenocytes stimulated with the 15-mer peptide pool. The splenocytes were harvested 14 days after the third immunization with EXCT4-mgG-2, EXCT4-mgG-2(−N − O) or mock and stimulated with the peptide pool overnight. **(E)** IgG1 and IgG2c levels in serum samples collected 14 days after the third intra muscularly (i. m.) immunization with EXCT4-mgG-2 or EXCT4-mgG-2(−N − O). **(F)** Serum antibody reactivity against EXCT4-mgG-2 and EXCT4-mgG-2(−N − O) was assessed in serum samples collected 14 days following immunization with EXCT4-mgG-2 or EXCT4-mgG-2(−N − O). ^+^ For immunization group EXCT4-mgG-2(−N − O), the antibody reactivity was corrected by subtracting values corresponding to glycosidase-specific binding in serum from mice immunized with the glycosidase enzymes in absence of EXCT4-mgG-2. SFC = Spot forming cells. Statistical analysis was performed with Saphiro-wilk test and Tukey´s multiple comparison test **(A-D)**, Kruskal-Wallis test **(E)** or Mann-Whitney test **(F)**. Values are expressed as means ± SEM.

To investigate the T-cell responses, spleens were harvested 10 days after the final immunization of C57BL/6 mice, and the splenocytes were stimulated with a peptide pool consisting of overlapping 15-mers spanning the entire EXCT4-mgG-2 protein sequence. The cells were then subjected to fluorospot analysis, which showed that interferon gamma (IFN-γ), interleukin 2 (IL-2) and tumour necrosis factor alpha (TNF-α) were significantly more expressed in splenocytes from EXCT4-mgG-2-immunized mice compared to those from EXCT4-mgG-2(−N − O)-immunized mice (Fig 3A). Moreover, the frequency of splenocytes expressing all three cytokines simultaneously was higher in the EXCT4-mgG-2 group (Fig 3B). Splenocytes from mice immunized with EXCT4-mgG-2(−N − O) also showed elevated levels of all three cytokines compared to the mock immunized mice (Fig 3A and 3B). Next, splenocytes were stimulated with the intact glycosylated EXCT4-mgG-2 antigen and subjected to fluorospot analysis. Splenocytes from both immunized groups, EXCT4-mgG-2 and EXCT4-mgG-2(−N − O), showed higher expression of both INF-γ and IL-2 compared to the mock controls (Fig 3C). Notably, only IL-2 expression was significantly higher in EXCT4-mgG-2 group compared to the EXCT4-mgG-2(−N − O) group. Then, splenocytes were stimulated with the 15-mer peptide pool, and intracellular INF-γ expression in CD4+ and CD8 + T cells was assessed by flow cytometry (S4 Fig). A fraction of CD8 + T cells from mice immunized with EXCT4-mgG-2 and EXCT4-mgG-2(−N − O) showed intracellular staining for INF-γ, compared to CD8 + T cells from the mock immunized mice (Fig 3D). Only CD4 + T cells from mice immunized with the EXCT4-mgG-2 antigen demonstrated intracellular INF-γ expression (Fig 3D). When combining both CD4+ and CD8 + T cells responses, only the EXCT4-mgG-2 immunized group exhibited a clearly increased number of INF-γ reactive cells (Fig 3D). We next determined if the CD4 + T cell response induces a glycosylation dependent antibody response against EXCT4-mgG-2. Serum samples were collected 14 days following the third intramuscular immunization with EXCT4-mgG-2 or EXCT4-mgG-2(−N − O) and the IgG1 and IgG2c levels were determined. Immunization with EXCT4-mgG-2 generated lower titres of IgG1 compared to EXCT4-mgG-2(−N − O) while there was no difference in IgG2c titres (Fig 3E) corroborating that glycosylation is important for mounting a Th1 response. Furthermore, the serum samples were investigated for reactivity towards EXCT4-mgG-2 and EXCT4-mgG-2(−N − O) in an ELISA. Antibodies in serum from EXCT4-mgG-2(−N − O) immunized mice showed reduced reactivity towards EXCT4-mgG-2, while antibodies in serum from EXCT4-mgG-2 immunized mice equally well recognized both EXCT4-mgG-2 and EXCT4-mgG-2(−N − O) (Fig 3F).To compensate for the antibody reactivity against the glycosidases used in the deglycosylation of EXCT4-mgG-2(−N − O), mice were immunized with an injection fluid containing the glycosidases but lacking EXCT4-mgG-2. Sera from these mice were analysed by ELISA for reactivity against EXCT4-mgG-2 and EXCT4-mgG-2(−N − O) (S5 Fig). The mean antibody titres obtained from these control animals were subtracted from the titres of mice immunized with EXCT4-mgG-2(−N − O), depicted in Fig 3F. Taken together, it appears that CD4 + T cells can differentiate between epitopes within mgG-2 depending on the glycosylation status, indicating glycosylation dependent recognition by CD4 + T cells that can inactivate important functions of the HSV-2 associated mgG-2 upon subsequent infection, thereby preventing neuronal spread.

### The HSV-2_ΔmgG-2_ mutant produces sgG-2

To further explore the function of mgG-2 in facilitating neuronal spread we utilized HSV-2_WT_, HSV-2_ΔmgG-2_ and HSV-2_rescue_ viral strains (Fig 1A and 1B). First, the production of sgG-2 for HSV-2_ΔmgG-2_, HSV-2_rescue_, and HSV-2_WT_ was determined. African green monkey kidney cells (GMK-AH1) were infected at a multiplicity of infection (MOI) 1 of respective viral strain, and the supernatants were collected when complete cytopathic effect was observed. The supernatants were normalized to the same amount of produced virus (PFU/mL). Western blot analysis indicated as expected that sgG-2 (44 kDa) are produced for all three HSV-2 strains (S6 Fig). The lack of expression of mgG-2 in the HSV-2_ΔmgG-2_ viral strain was previously verified [34].

### HSV-2_ΔmgG-2_ infects vaginal tissue but presents low mortality and disease scores

The natural course of intravaginal inoculation of HSV-2 has been described in detail [39]. Briefly, free virus particles enter the mucosal epithelial cells, replicate in the mucosa, followed by further spread to the lamina propria, autonomic neurons, blood, lymph nodes and to free nerve endings in the mucosa and lamina propria. Thereafter virus is transported in retrograde direction to the lumbar dorsal root ganglia and then proceeds to the spinal cord followed by encephalitis and death. The viral titer in vaginal washes after infection in unvaccinated mice correlate well with spread to ganglia and spinal cord.

**Fig 4:**
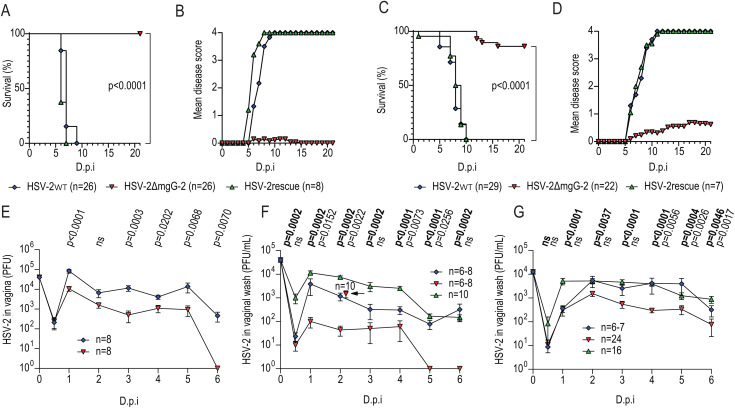
HSV-2_∆__mgG-2_ infection induces low genital disease scores and low mortality. **In addition, HSV-2**_**∆mgG-2**_ **infects and replicate in the genital tract but show impaired release from the surface of vaginal epithelial cells. (A-B)** C57BL/6 mice were infected intravaginally with 10 x LD_50_ of HSV-2_WT_, HSV-2_∆mgG-2_ or HSV-2_rescue_. The survival rate and disease score were followed until day 21. **(C-D)** DBA/2 mice were infected intravaginally with 125 x LD_50_ of HSV-2_WT_, HSV-2_∆mgG-2_ or HSV-2_rescue_. **(E)** Vaginas from C57BL/6 mice were excised at different timepoints after genital infection with 10 x LD_50_ of HSV-2_WT_ or HSV-2_∆mgG-2_ and HSV-2 plaque forming units (PFU) were assessed. **(F)** The PFU content in vaginal washings of C57BL/6 infected mice. In 10 C57BL/6 mice the infective dose was increased to 25 x LD50 PFU and PFU content was calculated at day 2 post infection (marked with an arrow). **(G)** The PFU content in vaginal washings from DBA/2 mice infected with 125 x LD_50_ of HSV-2_WT_, HSV-2_∆mgG-2_ or HSV-2_rescue_. PFUs (detection limit 40 PFU/mL) are expressed as means ± SEM. Statistical analysis is performed with pairwise Log-rank (Mantel-Cox) test **(A and C)** or Mann-Whitney test **(E-G)**. P-values are calculated for HSV-2_rescue_ versus HSV-2_∆mgG-2_ indicated in bold and for HSV-2_WT_ versus HSV-2_∆mgG-2_ indicated in normal script **(F and G)**. Data are calculated from two to three independent experiments. D.p.i. - days post infection.

Consequently, C57BL/6 mice were infected intravaginally with 10 x LD_50_ of HSV-2_WT_, HSV-2_ΔmgG2_ or HSV-2_rescue_. Survival (Fig 4A) and disease score (Fig 4B) were observed for 21 d.p.i. In mice infected with HSV-2_WT_ or HSV-2_rescue_, symptoms appeared 6 d.p.i. and presented a progressive genital and systemic infection and were all euthanized. In contrast, all mice infected with HSV-2_ΔmgG-2_ survived (Fig 4A) and presented no or limited inflammation in vagina, vulva and perineal region reflected by low disease scores (Fig 4B). Ten C57BL/6 mice infected with a higher dose of HSV-2_ΔmgG2_ exhibited viral loads in vaginal washings comparable to those of mice infected with HSV-2_WT_, despite this all mice survived until 21 d.p.i (Fig 4F and S3 Table). No clinical signs of spread to the autonomic neurons (constipation and urine retention) were observed in the mice infected with HSV-2_ΔmgG-2_. Next, the more HSV-2 susceptible DBA/2 mice were infected with 125 x LD_50_. The outcome of the infection with HSV-2_WT_ and HSV-2_rescue_ was similar, and all mice were euthanized (Fig 4C and 4D). After infection with HSV-2_ΔmgG-2_, 25 of 29 (86%) mice survived. Although four mice succumbed, the infection was more prolonged, and the mice were euthanized between 12 and 16 d.p.i. (Fig 4C and 4D). To exclude that the HSV-2_ΔmgG-2_ had reverted to the HSV-2_WT_ phenotype, spinal cord DNA from one euthanized DBA/2 mouse was prepared for HSV-2 gG-2 gene sequencing as described [40]. The sequence presented the identical frameshift mutation in the mgG-2 gene as described for HSV-2_ΔmgG-2_. In conclusion, HSV-2_ΔmgG-2_ shows greatly reduced capacity to promote genital and neurological disease.

Next, C57BL/6 mice were infected with 10 x LD_50_ of HSV-2_WT_ or HSV-2_ΔmgG-2_ and the vagina was excised 12 h.p.i. or 1, 2, 3, 4, 5 or 6 d.p.i. For both HSV-2_WT_ and HSV-2_ΔmgG-2_ the number of infectious particles declined approximately 100 times, compared to the input levels, at 12 h.p.i. (Fig 4E). The peak in viral load was reached 24 h.p.i. for both strains. At 6 d.p.i. with HSV-2_ΔmgG-2_ no virus was detectable and HSV-2_ΔmgG-2_ presented lower viral loads at all time points from day 1 and onwards.

The HSV-2 infection was estimated in vaginal washes at the same time-points after infection also including the HSV-2_rescue_ strain. Although at lower levels, similar kinetics was described as for the vaginal infection. The input virus was readily cleared within 12 h.p.i. and the peak in viral load was obtained 1 d.p.i. (Fig 4F). The differences between HSV-2_rescue_ and HSV-2_ΔmgG-2_ were statistically significant for all time points after infection. In cultured primary vaginal epithelial cells derived from C57BL/6 mice, HSV-2 was shown to be shed mostly from the apical surface [41]. The viral load in the vaginal washes can therefore be used as marker of release of extracellular HSV-2 in the vaginal infection before virus infected epithelial cells are sloughed off. Based on the area under curve (AUC) method for estimation of the viral load between day 1 and day 2, HSV-2_WT_ produced 7.6 times more PFU as compared with HSV-2_ΔmgG-2_ in vaginal tissue while in vaginal washes the difference was 77 times (p = 0.01). Estimating viral load for 1–6 d.p.i. using AUC showed that HSV-2_WT_ produced 7.0 times more PFU in vaginal tissue and 17.9 times more PFU/mL in vaginal wash compared to HSV-2_ΔmgG-2_ (Fig 4E and 4F).

The more sensitive DBA/2 mice were infected with 125 x LD_50_, and infectious particles were measured in the vaginal washes (Fig 4G). The sum of mean values of infectious particles for 12 h.p.i. and 1–6 d.p.i. in the vaginal washes was 29,223 PFU for HSV-2_WT_, 39,626 PFU for HSV-2_rescue_, and 5,945 PFU for HSV-2_ΔmgG-2_. The differences between HSV-2_rescue_ and HSV-2_ΔmgG-2_ were statistically significant for days 1–6. As the production of infectious viral particles was measured only in vaginal washes no ratio between PFU in vagina versus vaginal washes could be calculated.

### Reduced spread of HSV-2_ΔmgG-2_ in serum, genital lymph nodes and neuronal tissue

**Fig 5:**
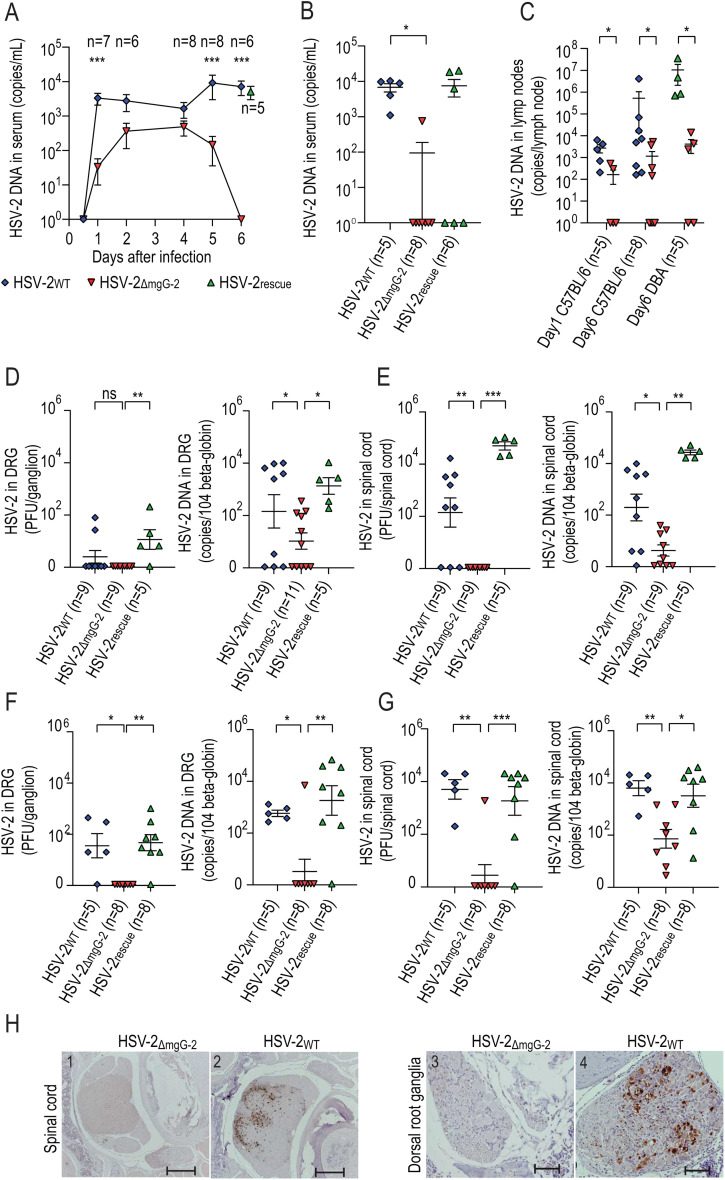
Assessment of the spread of HSV-2 to blood, genital lymph nodes, dorsal root ganglia and spinal cord, showing impaired capacity for neuronal spread of HSV-2_∆__mgG-2_. **(A)** C57BL/6 mice were infected with 10 x LD_50_ of HSV-2_WT_ or HSV-2_∆mgG-2_, and HSV-2 DNA was calculated at 1-6 d.p.i. in serum. Sera from 5 C57BL/6 mice infected with the HSV-2_rescue_ were analysed at day 6 (marked in green) **(B)** DBA/2 mice were infected with 125 x LD_50_ of HSV-2_WT_, HSV-2_∆mgG-2_ or HSV-2_rescue_, and HSV-2 DNA content in serum was calculated at 6 d.p.i. **(C)** C57BL/6 mice were infected with 10 x LD_50_ of HSV-2_WT_ or HSV-2_∆mgG-2_ and HSV-2 DNA was calculated in genital lymph nodes at 1 or 6 d.p.i. **(D-E)** C57BL/6 mice were infected with 10 xLD_50_ and **(F-G)** DBA/2 mice were infected with 125 x LD_50_ of HSV-2_WT_, HSV-2_∆mgG-2_ or HSV-2_rescue_. Infectious HSV-2 and HSV-2 DNA copy numbers were analysed 6 d.p.i. (D and F) per single dorsal root ganglion and (E and G) in the whole spinal cord. **(H)** Paraffin sections from the vertebral column of DBA/2 mice 6 d.p.i. infection with (panel 1 and 3) HSV-2_∆mgG-2_ or (panel 2 and 4) with HSV-2_rescue_. HSV-2 antigens (brown) were visualized using a polyclonal rabbit anti-HSV-2 serum. Panel 1-2 show the spinal cord at the thoracic level and panel 3-4 show the dorsal root ganglia at the lumbosacral level from the same animal. After infection with HSV-2_rescue_ several neurons and glial cells in the dorsal horns and neurons and satellite cells in the ganglia are infected. Bars: 1-2; 500 μm, 3-4; 100 μm. HSV-2 DNA copies are expressed per mL serum (detection limit 100 copies) or per lymph node (detection limit 80 copies). Values are expressed as means ± SEM. HSV-2 DNA copy numbers were standardized to 10^4^ beta-globin genes. The virus detection limit was 40 PFU or 160 HSV-2 DNA copies per 3 ganglia or whole spinal cord. Statistical analysis is performed with Mann-Whitney test. Data are calculated from one to two independent experiments for each mouse strain except for the immunohistochemistry data where one experiment representative of the two performed is shown. D.p.i. - days post infection.

After infection of C57BL/6 mice with 10 x LD_50_ of HSV-2_WT_ the viral DNA genome copy numbers in serum reached a plateau 1 d.p.i., which lasted until 4 d.p.i. whereafter the viral load increased almost 10 times (Fig 5A). In contrast, after infection with 10 x LD_50_ of HSV-2_ΔmgG-2_ viral DNA increased more slowly and reached lower levels as compared with the HSV-2_WT_ and was cleared 6 d.p.i. Viral load was also measured after infection with HSV-2_rescue_, which presented similar values as for HSV-2_WT_ 6 d.p.i. (Fig 5A). These data show, also including results from the vaginal infection, that there is a clear difference in the kinetics of the infection between HSV-2_WT_ and HSV-2_ΔmgG-2_. Thus, in the initial stage up to day 4–5, the difference in the viral load is moderate, while after this period the infection with HSV-2_ΔmgG-2_ is rapidly cleared. In DBA/2 mice, HSV-2 DNA was measured in serum 6 d.p.i. Similar levels were detected for HSV-2_WT_ and for HSV-2_rescue_ as described for the C57BL/6 mice. One DBA/2 mouse of eight, infected with HSV-2_ΔmgG-2_, presented a value of 747 viral DNA copy numbers per mL serum (Fig 5B).

In C57BL/6 infected mice, viral DNA was detected in genital lymph nodes 1 d.p.i. with an increase of the viral load for HSV-2_WT_ to 4 x 10^5^ viral DNA copy numbers 6 d.p.i., as compared with 1 x 10^3^ copy numbers after infection with HSV-2_ΔmgG-2_. In DBA/2 infected mice, the mean viral load per lymph node 6 d.p.i. for HSV-2_rescue_ was 1 x 10^7^ HSV-2 DNA copy numbers as compared with 5 x 10^3^ after infection with HSV-2_ΔmgG-2_ (Fig 5C).

Next, C57BL/6 mice were infected with 10 x LD_50_ and DBA/2 mice with 125 x LD_50_ of HSV-2_WT_, HSV-2_ΔmgG-2_ and HSV-2_rescue_. DRG and spinal cord were collected at 6 d.p.i., infectious virus was assayed by a plaque assay and HSV-2 DNA was quantified by real-time PCR. In addition, spinal cord and ganglia were collected at 14 or 21 d.p.i. from five mice infected with HSV-2_ΔmgG2_. For most animals infected with HSV-2_WT_ or HSV-2_rescue_, viral DNA was detectable in both the DRG and the spinal cord 6 d.p.i. (Fig 5D and 5E). The most important finding which explains the high survival rate of mice after infection with HSV-2_ΔmgG-2_, was that the viral load in DRG and spinal cord was significantly reduced. After infection of C57BL/6 mice with HSV-2_ΔmgG-2_ no infectious viral particles were detected in DRG and spinal cord (Fig 5D and 5E and S4 Table). After infection of DBA/2 mice with HSV-2_ΔmgG-2_, infectious virus was only detected in the spinal cord in one mouse and significantly lower values HSV-2 DNA were detected in both DRG and spinal cord as compared with infection with HSV-2_WT_ or HSV-2_rescue_ strains (Fig 5F and 5G). The lack of infectious HSV-2 and production of viral proteins in DRG and spinal cord after infection with HSV-2_ΔmgG-2_ were also confirmed with an immunohistochemistry technique using a rabbit polyclonal anti-HSV-2 serum for detection (Fig 5H).

## Discussion

In this study, we demonstrate that mgG-2 is a critical determinant of HSV-2 dissemination to neuronal tissues in vivo and that its glycosylation plays a key role in shaping protective immune responses. Using a combination of infection models and vaccination studies, we show that loss of mgG-2 impairs viral spread to dorsal root ganglia and the central nervous system, while mgG-2 elicits Th1-biased CD4 + T cell responses associated with protection when administered as a glycosylated antigen for immunization. These findings provide new insight into both the role of mgG-2 in HSV-2 pathogenesis including neurotropism, and the importance of glycosylation of this protein for vaccine efficacy.

Previous studies have established that gG-2 is dispensable for viral replication in vitro but can influence viral egress and interactions with glycosaminoglycans [34, 42] but the role of mgG-2 in viral dissemination in vivo has remained largely unexplored. We and others have shown that glycosylation of viral glycoproteins, including those of herpesviruses, can modulate immune recognition and antigenicity, this post-translational modification is therefor most likely is of importance for vaccine development [43, 44, 45, 46, 47].

Here, using a genital infection model, we show that HSV-2_ΔmgG-2_ replicates in the vagina; however, viral spread from vagina to the blood, genital lymph nodes, sensory ganglia, spinal cord, and genital autonomic neurons, was severely impaired in the absence of mgG-2. Following infection with HSV-2_ΔmgG-2_, the number of infectious viral particles in the vaginal lumen was initially reduced 10 times compared to HSV-2_WT._ These observations agree with impaired release of infectious HSV-2_ΔmgG-2_ particles in mouse fibroblast L-cell cultures where extracellular viral particles were approximately 10 times lower as compared with HSV-2_WT_ [42]. However, when the infective dose of HSV-2_ΔmgG-2_ was increased, the content of infectious HSV-2 in the vaginal washes was similar at day 2 as for HSV-2_WT_. Despite this increase, neuronal spread was severely reduced or absent, suggesting that impaired spread of virus from infected vaginal epithelia to sensory neurons due to lack of mgG-2 was the most important explanation.

In the initial infection HSV uses gB, gC and gD to attach to cellularly produced glycosaminoglycans (GAGs) on the cell membrane. After attachment, the gD binds to a cell receptor for entry. In murine keratinocytes nectin-1 was shown to be the primary receptor [48]. Using knock-out C57BL/6 mice lacking the cellular receptor herpes virus entry mediator (HVEM) or nectin-1 or both, Tayler et al., showed that HVEM or nectin-1 was required for infection of the vaginal epithelium after genital infection but neither of the receptors were essential for spread of HSV-2 to DRG and spinal cord [49]. After entry and replication, progeny virus is released by exocytosis as individual virions [50, 51]. The last phase of the infection is the release of infectious virions from infected cells for further spread. To avoid that GAGs block the release of virions by binding to the HSV attachment proteins, HSV has developed counteractive strategies. For example, Hadigal et al., showed in HSV-1 infected cells that the levels of cell membrane bound heparan sulfate (HS) GAGs were substantially decreased by up-regulation of the host enzyme heparanase-1, cleaving the HS on the cell surface [52]. In an earlier report, HSV-2_ΔmgG-2_ was shown to be impaired in egress, and mature infectious viral particles that accumulated on cell infected membranes in different cell lines could effectively be released using GAG-mimicking oligosaccharides. A conclusion was that mgG-2 balances the interaction between the virion and GAGs present at virus infected cell membranes to enable egress [42]. In the present study HSV-2_ΔmgG-2_ showed impaired egress from vaginal epithelial cells into other compartments, including the nervous system, which could contribute to the high survival rate after genital infection with HSV-2_ΔmgG-2_. Further studies will address whether mgG-2 also has direct functions in the neuronal infection, for example by facilitating entry into the nerve endings. The observed impairment in cellular egress suggests that HSV-2 lacking mgG-2 may also be compromised in its dissemination to peripheral tissues beyond ganglia and spinal cord. However, this cannot be confirmed, as viral load was not assessed in tissues outside those relevant to the natural course of intravaginal inoculation described by Parr and Parr [39].

As mentioned, another aspect with relevance for vaccine development is the impact of post translational glycosylation of viral surface proteins. To address the knowledge gap regarding the impact of glycosylation of the putative mucin like domain of mgG-2, we defined the entire glycosylation profile of EXCT-4-mgG-2, the recombinantly expressed mgG-2. Our detailed characterization of the glycan content of EXCT4-mgG-2, contradict the notion that mgG-2 holds densely clustered O-linked glycans in its predicted mucin-like region. When the protein is recombinantly expressed in CHO-cells, 12 dispersed sites harbour O-linked glycans, which partially agrees with previous work by Iversen et al [23]. The number of identified glycosites were in the same range but there was only partial overlap when considering the individual glycosites. The discrepancy in glycan occupancy can possibly be attributed to cell specific differences in expression of glycosyltransferases, e.g., CHO-cells lack α2–6 sialyltransferase expression, but more importantly exhibit limited expression of a subset of GalNAc-transferases, that could negatively affect site occupancy in addition to alterations in glycan structure heterogeneity [53, 54].

Based on the glycan profile we constructed several glycan-modulated vaccine candidates and showed that glycosylation of EXCT-4-mgG-2 contributes to generating a more efficient humoral immune response upon immunization. With the amino acid backbone of the recombinant antigens being identical, we attribute the reduced survival of the EXCT4-mgG-2(−N − O) immunized mice to the absence of glycosylation. In contrast, removal of N-linked, O-linked, or sialic acid glycan structures alone, presented no effect on either mortality or morbidity. Moreover, we showed that immunization with EXCT4-mgG-2 elicited a robust adaptive immune response including Th1-polarized cells that produced IL-2 and INF-γ, as compared to immunization with EXCT4-mgG-2(−N − O), suggesting enhanced T helper cell function that can contribute to B cell help and class switching in favour of IgG2c. This is interesting since glycans can, either on their own or in combination with the protein backbone, constitute B- and T- cell epitopes and thereby enhance antigenicity [55]. While we could not identify O-linked glycosylation at threonine 504 in EXCT4-mgG-2, the position previously shown to be immunodominant in a glycosylation dependent manner [18], we showed that the overall reactivity of serum from immunized mice was glycan dependent. This suggests that the glycans may contribute directly to glycoepitope formation, however, the fact that spleenocytes from EXCT4-mgG-2-immunized mice showed a stronger response to the unglycosylated peptide pool makes it difficult to reconcile with the presence of T-cell specific glycoepitopes. The dampened response towards the whole glycosylated antigen might be explained by delayed display by the antigen-presenting cells (APCs) as the whole antigen must be processed into shorter peptides before it can be integrated into the MHC-peptide complex, although the observed effect should be interpreted with caution since the splenocytes were only incubated with the antigen for 42 hours, according to a protocol designed for peptide stimulation. Alternative explanations to the superior efficiency of EXCT4-mgG-2 could be: (i) that the glycans focus the response towards immunodominant epitopes in unglycosylated peptide stretches of the antigen, as was recently suggested for HSV-1 glycoprotein B [56], (ii) that the glycans affect antigen uptake by interaction with glycan-binding C-type lectin receptors on APCs and thereby facilitating a more efficient antigen presentation [57]. These possible explanations are not mutually exclusive and thus could act synergistically to generate the enhanced IL-2 and INF-γ response.

In the context of HSV-2 infection, such enhanced immune responses may limit viral replication at the site of entry and thereby reduce the likelihood of viral spread to sensory nerve endings and subsequent neuroinvasion. Glycosylation of EXCT-4-mgG-2 may contribute to this protective effect by modulating antigen processing and presentation, resulting in more efficient priming of CD4 + T cells. In addition, glycan structures may influence epitope accessibility and immune focusing, potentially enhancing recognition of protective epitopes that drive Th1-biased responses. Importantly, the enhanced Th1-polarized CD4 + T cell responses observed following immunization with glycosylated mgG-2 provide a plausible mechanistic link to the reduced viral dissemination to neuronal tissues. CD4 + T cells producing IFN-γ are known to play a critical role in controlling herpesvirus infections by promoting antiviral states in infected epithelial cells and supporting the clearance of infected neuronal cells [58]. Together, these mechanisms suggest that glycosylation of mgG-2 not only enhances immunogenicity but also shapes the quality of the immune response in a manner that is particularly effective at limiting viral spread to dorsal root ganglia and the central nervous system. Furthermore, Görander et al. have, using INF-γ-gene knockout mice (KO) on a C57BL/6 background, shown that INF-γ produced by CD4 + T cells are essential to generate protection after immunization with glycosylated native mgG-2 followed by genital challenge [33]. In addition, B cell KO mice, immunized with glycosylated mgG-2, presented lower survival rate and higher vaginal viral titers, as compared with vaccinated B-cell KO mice which received passive transfer of immune serum from vaccinated C57BL/6 mice [59].

Due to the high prevalence and morbidity of HSV-2 infection there is a great need both for a therapeutic and a prophylactic vaccine. Unfortunately, no vaccine has been approved despite great efforts. The major challenge has been that promising results after vaccination in mice and guinea pigs has only in part been translated and replicated in clinical trials. In the initial preclinical development of a vaccine antigen against HSV-2 infection, experiments in mouse models are often followed by vaccination and genital challenge in guinea pigs since this model mimics the human disease with recurrences and viral shedding. Several studies using recombinant gD-2 antigen with different adjuvant in the guinea pig model have showed induction of neutralizing anti-gD-2 antibodies, significantly higher survival, lower acute disease score, lower viral load in ganglia and spinal cord and reduced recurrent disease. However, none of the vaccines were able to completely block mucosal infection (sterilizing immunity) as well as the following recurrent shedding [60–63, 64]. Human clinical trials using adjuvanted gD showed partial protection against HSV-1 and protection against HSV-2 in seronegative women, but failed to demonstrate consistent efficacy across broader clinical populations [65, 66].

The discrepancies between the promising results in animal vaccine studies and the modest result in clinical studies is challenging. Of importance is that HSV-1 and HSV-2 have co-evolved with humans for a long time. HSV has therefore developed human specific immune evasion strategies which cannot be recapitulated in animal models [67]. For example, [68] analysed the specificity of elicited anti-gD-2 antibodies to adjuvanted gD-2 in the latest prophylactic vaccine phase III clinical trial HerpeVac (GlaxoSmithKline). The authors showed that antibodies identifying three crucial linear epitopes in gD-2 with importance for entry and cell to cell spread were lacking, while such antibodies are induced and protective in mice and guinea pigs. We conclude that none of the vaccination animal models have so far predicted the outcome in clinical studies.

A complementary approach to animal models is the study of the immune responses to mgG-2 in already HSV-2 infected individuals. Although mgG-2 is dispensable for HSV-2 replication in cell cultures, Liljeqvist et. al., demonstrated that mgG-2 negative clinical HSV-2 isolates are rarely detected. Among 2 400 clinical HSV-2 isolates tested using an anti-mgG-2 monoclonal antibody, only two HSV-2 isolates were truly mgG-2 negative, i.e., lack of mgG-2 due to a frameshift mutation and lack of anti-mgG-2 antibodies in patient serum [40].

In another study, Eriksson et. al. showed that HSV-1 seronegative and recurrent symptomatic HSV-2 seropositive individuals responded with significantly lower Th1 cytokine production (IFN-γ, IL-2 and TNF-α) as compared to HSV-1 negative and asymptomatic HSV-2 infected individuals after stimulation with sgG-2 or mgG-2 [26]. We have characterized the antibody responses to EXCT4-mgG-2 antigen in HSV-1 and HSV-2 infected patients and compared to the antibody responses to recombinantly produced gD-2 antigen. We found that the median concentration of anti-mgG-2 antibodies was five times lower in HSV-1 + 2-infected subjects as compared with cross-reactive anti-gD-1 and anti-gD-2 antibodies, and three times lower in HSV-2 infected subjects as compared with anti-gD-2 antibodies. Furthermore, the anti-EXCT4-mgG-2 antibodies presented no neutralization capacity but ADCC by human granulocytes, monocytes and NK cells, results which are promising for a therapeutic vaccine with the aim to boost the immune responses to mgG-2 in already HSV-2 infected subjects [69]. We speculate that a prophylactic mgG-2 vaccine against HSV-2 infection may be favourable, not only due to the novel finding that it is important for preventing neuronal spread, but also because the protein elicits only type-specific HSV-2 immune responses avoiding interference at immunization of cross-reactive immune responses from an earlier HSV-1 infection.

To achieve a functional therapeutic vaccine in already HSV-2 infected subjects, the objective is to reduce clinical lesions and importantly, inhibit or reduce reactivation and thereby preventing both symptomatic and asymptomatic shedding of HSV-2. This task is more difficult as compared to prophylaxis because the virus has already established latency in sensory ganglia. However, there is an approved vaccine (Shingrix) against reactivation of VZV, a herpesvirus which also establish latency in sensory ganglia. The adjuvanted vaccine use the glycoprotein E produced in CHO cells and prevent herpes zoster in older populations (≥ 50 years of age). The vaccine efficacy was impressively between 96.6% and 97.9% for all age groups [70]. This is the first example of a vaccine able to prevent reactivation from the sensory ganglia of an *Alphaherpesvirinae* member. Notably, in this context, we have previously demonstrated that the CHO cell–derived glycoprotein E exhibits less dense glycosylation compared to the human cell–derived counterpart, suggesting that glycan composition and density can directly influence vaccine efficacy [71, 46].

An important question is to what extent the glycosylation-dependent immune responses observed in this study are conserved in humans. Although our findings are based on a murine model, several lines of evidence suggest that the underlying mechanisms may be translatable. Glycosylation is known to influence antigen processing, epitope presentation, and immune recognition across species, and similar roles for glycans in shaping antibody and T cell responses have been described for several human viral pathogens including herpesviruses [43, 44, 45, 46, 47]. Together, these observations suggest that preserving functionally relevant glycosylation patterns of mgG-2 will be important for inducing optimal immune responses in humans. In the context of vaccine development, this highlights the need to carefully consider expression systems and glycoengineering strategies to ensure that recombinant antigens retain appropriate glycan structures. More broadly, our findings support the concept that glycosylation is not merely a structural feature of viral proteins but a critical determinant of vaccine efficacy, which should be considered in the design of next-generation HSV-2 vaccines targeting mgG-2.

A limitation of this study is that we did not identify individual B and T cell epitopes and could therefore not assess the impact of individual glycan structures on the above-mentioned effects on the adaptive immune response. Also, in this work, deglycosylated variants of EXCT4-mgG-2 were obtained by enzymatic treatment, and it is expected that residual glycan structures are present on a fraction of the proteins. Especially the O-linked glycans, which require multiple rounds of enzymatic treatments to remove, may be incompletely deglycosylated. Consequently, it cannot be ruled out that the partial protective effect observed in mice immunized with EXCT4-mgG-2(−N − O) comes from incompletely deglycosylated EXCT4-mgG-2. Thus, it is possible that this complicates the interpretation of the results from the adaptive immune experiments, including antibody levels and reactivity as well as the T cell responses. However, when comparing the survival rate of mice immunized with EXCT4-mgG-2(−N − O) antigen with the results from the infection experiments using the HSV-2_WT_ and HSV-2_ΔmgG-2_ strains we conclude that, if any, an incomplete deglycosylation would rather overestimate the survival rate of the mice immunized with EXCT4-mgG-2(-N-O). Attempts were made to produce recombinantly expressed EXCT4-mgG-2 in an *in vitro* translation *E. coli* system that would generate a protein devoid of glycan structures corresponding to EXCT4-mgG-2(-N-O), but, despite great efforts, isolation of the protein failed. The failure to isolate secreted protein suggests that the glycans are necessary for proper protein processing. Absence of glycans may cause misfolding of the protein during its transport through the secretory pathway causing premature degradation of EXCT4-mgG-2 in this expression system [72].

In conclusion, earlier vaccine studies have focused mostly on gD-2 and gB-2, proteins which are essential for entry and fusion of the viral particle infecting the cell, but which failed as antigens in clinical trials. We show here that mgG-2 is important for viral egress from infected epithelial cells and spread to the neurons in an infection model with an HSV-2 mgG-2 negative mutant. Moreover, vaccination using EXCT4-mgG-2 as antigen together with adjuvant elicited immune responses which impaired the egress function of mgG-2 in wild-type HSV-2 used for challenge. These observations present a novel approach for development of an HSV-2 vaccine. Future studies should aim to define the specific T cell epitopes within mgG-2 and determine how the glycosylation profiles influence antigen processing and presentation. In addition, structural and biochemical analyses to identify potential interaction partners of mgG-2 could provide further insight into its role in viral dissemination. Finally, evaluating glycosylated mgG-2 vaccine candidates in models that more closely mimic human infection, as well as assessing immune responses in human cells or clinical samples, will be important for translating these findings into vaccine development.

## Materials and methods

### Ethics statement

The animal studies were approved by the Swedish board of agriculture (Dnr 5.8.28-18516/2018), (Dnr 5.8.18-08670/2022) and (Dnr 6054–2021).

### Cells and viruses

GMK-AH1 were cultured in Eagle’s minimal essential medium (EMEM, ThermoFisher) supplemented with 2% inactivated FCS and 1% penicillin-streptomycin (PEST). Stock viruses were produced by infecting GMK-AH1 cells without serum in roller bottles at a MOI of 0.5. After complete development of cytopathic effect, the cells and medium were frozen and thawed. The lysate was centrifuged for 10 min at 3000 x g and the supernatant was harvested and kept at − 80 °C. HSV-2 was quantified by a plaque assay on monolayers of GMK-AH1 cells. The titre of the HSV-2_WT_ was 2.0 x 10^7^ PFU/mL, for HSV-2_ΔmgG-2_ 3.4 x 10^6^ PFU/mL, and for HSV-2_rescue_ 4.6 x 10^7^ PFU/mL. The HSV-2_WT_ strain 333 was a kind gift from the University of Pittsburgh 1993. The strain has been passaged <5 times at our laboratory. The GMK-AH1 cells were supplied by Karolinska Institutet in Stockholm. The cells were checked for Mycoplasma contamination by real-time PCR and for other bacterial and fungal contamination by culturing on agar plates. Contamination checks were performed at the Department of Clinical microbiology at the Sahlgrenska University hospital.

### Production of recombinant mgG-2

The recombinant mgG-2 construct (EXCT4-mgG-2) codes for amino acid A_345_-D_649_, comprising the extracellular region of mgG-2, V_667_-A_670_ from the transmembrane region and A_671_-D_698_, constituting the intracellular region of mgG-2, retrieved from the HSV-2_WT_ strain 333 (Fig 1A). The protocol is described in detail elsewhere [37]. Briefly, the CHO-K1 (028-W4) with the GS expression system was used (Lonza). 50 µM L-methionine sulfoximine (MSX) (Sigma Aldrich) was used as a selection agent for the transfected plasmid. The cells were kept under MSX selection pressure for 30 days before transferring to a 250 mL spinner flask (Bellco) containing 75 mL Dulbecco’s Modified Eagle Medium (L-glutamine-free, Gibco, ThermoFisher Scientific), 25 mL CD FortiCHO (ThermoFisher Scientific) and 50 µM MSX. Protein purification was performed using an ion exchange column (HiPrep QFF 16/10 (Cytiva) followed by a HiPrep 16/60 Sephacryl S-400 high resolution gel filtration column (Cytvia).

### Deglycosylation of EXCT4-mgG-2

For complete deglycosylation of EXCT4-mgG-2(−N − O), EXCT4-mgG-2 was incubated with 1 × glycobuffer 2, α2–3, 6, 8 neuraminidase (25 U/µg EXCT4-mgG2), O-glycosidase (20 000 U/µg EXCT4-mgG2), α-N-acetyl-galactosaminidase (10 U/µg EXCT4-mgG2) and PNGaseF (250 U/µg EXCT4-mgG2). Removal of only N-linked glycans from (EXCT4-mgG-2(-N) was performed using incubation with 1 × glycobuffer 2 and PNGaseF (250 U/µg EXCT4-mgG2). Removal of O-linked glycans from EXCT4-mgG-2(-O) was performed using incubation with 1 × glycobuffer 2, α2–3, 6, 8 neuraminidase (25 U/µg EXCT4-mgG2), O-glycosidase (20 000 U/µg EXCT4-mgG2) and α-N-acetyl-galactosaminidase (10 U/µg EXCT4-mgG2). Removal of sialic acids from EXCT4-mgG-2(-SA) was performed using incubation with 1 × Glycobuffer 2 and α2–3, 6, and 8 neuraminidases (50 U/µg EXCT4-mgG2). The enzyme control contained 1 × Glycobuffer 2, α2–3, 6, 8 neuraminidase (2500 U/mL), O-glycosidase (2 000 000 U/mL), α-N-acetyl-galactosaminidase (1000 U/mL) and PNGaseF (25 000 U/mL). All incubations were performed at 37 °C for 24 hours. All enzymes and glycobuffer 2 were purchased from New England Biolabs. Deglycosylation was confirmed using Western blot and lectin binding assay as described below. To remove free sugar residues, the protein preparations were dialyzed for 24 hours in PBS using the Slide-A-Lyzer Mini Dialysis Device 7K MWCO (ThermoFisher Scientific), with medium exchanges at hours 2, 4, 20 and 22.

Deglycosylation of EXCT4-mgG-2 was verified by western blot and lectin blot. In brief, the protein fractions were mixed with 2x SDS-PAGE sample buffer (25% 0.5M Tris-HCl, 20% SDS (10%), 40% glycerol, 10% 2-mercaptoethanol, 5% bromophenol blue) and heated to 95 °C for 10 minutes prior to separation on a NuPage Bis-tris 4–12% gel using MOPS running buffer (Invitrogen) at 100 V for 60 min using an EI9001-XCELL II Mini Cell (Novex) together with a Powerease 500 (Novex). The protein construct was transferred onto an Immobilon PVDF membrane in blotting buffer (NuPAGE Transfer Buffer; (ThermoFisher Scientific), 10% methanol). The membrane was blocked in blocking buffer (2% milk, 4% FCS) for 1 hour. As the primary antibody, a monoclonal mouse anti-mgG-2 antibody (O1.C5.B2 [28]) was used at a 1:2000 dilution, followed by incubation with a horse radish peroxidase-conjugated anti-mouse IgG (Aglient) at a 1:20 000 dilution. Bound antibodies were developed using SuperSignal West Dura Extended Duration Substrate (ThermoFisher Scientific) and detected in the ChemiDoc MP Imaging System (BioRad Laboratories).

Lectin blot was performed by loading, 2 µg each of EXCT4-mgG-2, EXCT4-mgG-2(−N − O), EXCT4-mgG-2(-N), EXCT4-mgG-2(-O) and EXCT4-mgG-2(-SA) to a NuPAGE Bis-Tris 4–12% gel (Invitrogen) and separated using MOPS buffer (Invitrogen) at 200 V. The separated proteins were transferred to a polyvinylidene difluoride membrane (Immobilon-FL 0.45 μm, (Merck Millipore)) using the SemiDry Transblot SD (Bio-Rad Laboratories). Membranes were blocked with block buffer (2% BSA Factor V (Sigma-Aldrich) and 0.1% Tween-20 (VWR chemicals) in PBS (Medicago) followed by incubation with biotinylated lectins; 3 µg/mL Concavalin A (Con A) (Vector Laboratories), 3 µg/mL Jacalin (Vector Laboratories) or 5 µg/mL Maackia Amurensis Lectin II (MAL II, Vector Laboratories) diluted in PBS-BSA-T, at 4 °C for 16–20 hours. Next, membranes were washed three times with 0.1% Tween-20 in PBS (Medicago) followed by incubation with Streptavidin-alkaline phosphatase diluted 1:2000 (Southern Biotech) for 1 h at 20–22 °C. Membranes were washed three times with 0.1% Tween-20 in PBS and developed using BCIP/NBT (Sigma Aldrich) for 1 minute.

### Sample preparation prior to LC-MS/MS

The recombinant EXCT4-mgG-2 samples (24 µg) were constituted in 0.1% sodium deoxycholate (SDOC) (Sigma Aldrich) in 50 mM triethylammonium bicarbonate (Sigma Aldrich) at 0.3 mg/mL. Reduction was done with 5 mM Tris(2-carboxyethyl)phosphine (Sigma Aldrich) at 58 °C for 20 min. The samples were then divided in two parts and digested with either pronase (Roche) or alpha-lytic protease (Sigma Aldrich) for 16 h at 37 °C, at protein:enzyme ratios of 1:20. SDOC was removed by acidification with trifluoroacetic acid followed by centrifugation and then desalted using peptide desalting spin columns (Pierce, ThermoFisher Scientific) according to the manufacturer´s protocol. The eluates were dried in a vacuum centrifuge and dissolved in 3% acetonitrile, 0.1% trifluoroacetic acid (Sigma Aldrich) prior to LC-MS/MS analysis.

### LC-MS/MS analysis

Nano-LC-MS/MS was done with the Orbitrap Exploris 480 mass spectrometer (ThermoFisher Scientific) interfaced with an Easy nLC 1200 liquid chromatography system. Analytes were separated using a trap column (15 cm x 0.1 mm, 5 μm particle size Acclaim Pepmap, ThermoFisher Scientific) and an in-house packed C18 analytical column (35 cm x 0.075 mm, 3 μm particle size (Dr. Maisch)) using an acetonitrile gradient in 0.2% formic acid during 90 min. Precursor ion mass spectra were acquired at 120 000 resolution with the scan range m/z 350–2000 and MS/MS analysis with a first mass of m/z 110. Higher-Energy Collisional Dissociation spectra of the most intense precursor ions were performed in data-dependent mode using 3s cycle times, a resolution of 30 000 and a maximum injection time of 100 ms. The normalized automatic gain control target was set to 150%. Normalized collision energies of 20% and 30% were applied on each precursor ion using an isolation window of two mass units. The exclusion time was 12 s. Each sample was run in triplicates using consecutive injections.

### LC-MS/MS data processing

Data analysis was performed with Proteome Discoverer version 2.4 (ThermoFisher Scientific) using the Byonic (Protein metrics) and Minora (ThermoFisher Scientific) feature detector nodes. The O-glycan modifications were set to with the addition of the HexNAc(2)Hex(2), HexNAc(2)Hex(2)NeuAc(2) and HexNAc(2)Hex(2)NeuAc(4) compositions. The number of allowed O-glycans per peptide was 0–2 of the same or with different compositions. The N-glycan modifications were set to `57 human plasma`. Additional allowed modifications were methionine oxidation and the Asn to Asp modification. Free cleavage sites were searched using the mgG-2 protein construct sequence, at a 5 ppm mass limit for the precursor ions and a 20 ppm mass limit for the MS2 ions. All glycopeptide identities were manually verified to contain the presence of the correct peptide and/or peptide+HexNAc ion and the proper set of oxonium ions. For instance, presence of the oxonium ion at m/z 274.09 for compositions including Neu5Ac, presence of m/z 366.14 for compositions including HexHexNAc and m/z 163.06 for compositions including oligomannose N-glycosylation. The maximum intensity measurement by Minora was used for the relative quantification measurements. In addition, manual inspection of the raw spectra using Xcalibur qual browser (ThermoFisher Scientific) was performed to confirm the presence of the N-linked glycoforms. The CVs for all glycoforms are based on the percental distribution within each LC-MS/MS injection, glycoforms with a relative abundance < 1% are excluded from the calculations.

Of note, the m/z value of the oxonium ions obtained during LC-MS/MS analysis is 204 for GlcNAc and GalNAc alike. Similarly, the addition of a Gal or Glc on the N-acetylated hexose (e.i GlcNAc or GalNAc) results in a peak at m/z 366 in the MS2 spectra. Hence, in our assay, it was not possible to distinguish between the types of hexoses. However, due to extensive previous glycoproteomic analysis of the HSV-2 and CHO-cell lines, done by us and others, and the confirmation of O-linked core 1 glycan structures, the m/z 204 and 366 peaks most likely correspond to GalNAc and GalGalNAc, respectively.

### Mouse model for immunization and HSV-2 genital challenge

The animals were kept at Experimental Biomedicine, Gothenburg University, the protocol was approved by the local ethics committee, and all procedures were performed according to approved guidelines and regulations. Female 6- to 8-week-old C57BL/6 mice were intramuscularly immunized three times at 10-day intervals using 2.5 µg EXCT4-mgG-2, EXCT4-mgG-2(−N − O), EXCT4-mgG-2(-N), EXCT4-mgG-2(-O), EXCT4-mgG-2(-SA) or controls (PBS alone). In all experiments adjuvant consisting of 250 µg alum (Alhydrogel) + 20 µg CpG (ODN1826, TCC ATG ACG TTC CTG ACG, TT), (Operon Biotechnologies GmbH) were used and diluted in 30 µL Tris-buffered saline (TBS). Six days prior to challenge, the mice were injected subcutaneously with 3 mg Depo-Provera (medroxyprogesterone acetate) (Pfizer). The mice were challenged intravaginally with 1 x 10^5^ PFU of HSV-2_WT_ (corresponding to 25 x LD_50_). Mice were scored daily for vaginal inflammation and disease and were graded as follows: 0 - no symptoms (healthy); 1 - genital erythema; 2 - moderate genital inflammation (blisters); 3 - severe symptoms or neurologic symptoms (purulent genital lesions, loss of hair); 4 - paralysis or generally poor condition. Mice presenting a score of 3 or higher were euthanized.

### Quantification of total IgG in mouse serum

Blood samples were drawn from the tail vein 14 days after the third immunization. Sera were separated from whole blood using centrifugation twice for 15 minutes at 400 g. ELISA plates (96-well) were coated with 100 µL (1 µg/mL diluted in carbonate buffer (pH 9.6)) EXCT4-mgG-2, EXCT4-mgG-2(−N − O), EXCT4-mgG-2(-N), EXCT4-mgG-2(-O) or EXCT4-mgG-2(-SA), for serum samples collected from mice immunized with EXCT4-mgG-2 (n = 29), EXCT4-mgG-2(−N − O) (n = 16), EXCT4-mgG-2(-N) (n = 10), EXCT4-mgG-2(-O) (n = 10) or EXCT4-mgG-2(-SA) (n = 10), respectively. Plates were blocked with 2% milk, and 100 µL of serum samples diluted 1:90, 1:270, 1:810, 1:2430, 1:7290, 1:21870, 1:65610, and 1:196830 in 1% milk + 0.05% Tween in PBS were added to the wells. The plates were incubated for 1.5 hours at 37 °C before washing with TBS that contained 0.05% Tween, followed by the addition of peroxidase-conjugated anti-mouse IgG (Sigma Aldrich) and incubation for 1.5 hours at 37 °C. Peroxidase activity was detected by the addition of o-phenylenediamine dihydrochloride (Sigma Aldrich), in citrate buffer. The reaction was stopped with sulfuric acid after 30 seconds, and the OD was measured at 490 nm in a microplate reader. The antibody titre was defined as the highest serum dilution giving an OD ≥ 0.250, defined as OD 0.2 above the value of the negative control.

### Serum IgG reactivity against recombinant mgG-2

96-well Maxisorp plates were coated with EXCT4-mgG-2 or EXCT4-mgG-2(−N − O) (1 µg/mL diluted in carbonate buffer (pH 9.6)) and incubated at 4 °C, overnight. Blocking of the plates was performed with 2% milk in PBS for 30 minutes at room temperature. Serum samples from mice immunized with EXCT4-mgG-2 or EXCT4-mgG-2(−N − O) was diluted 1:100 in dilution buffer (1% milk and 0.05% tween in PBS) and incubated for 1.5 h at room temperature followed by washing of the plates and addition of Alkaline phosphatase-conjugated anti-mouse IgG (Jackson ImmunoResearch Laboratories) diluted 1:1000 in dilution buffer. Following 1.5 hours incubation at room temperature the plates were again washed and 1 mg/mL p-nitrophenylphosphate (Medicago) dissolved in diethanolamine buffer was added. The plates were incubated 15 minutes in the dark before OD measurement at 405 nm in a microplate reader.

### Viral plaque assay

To measure viral replication in the genital tract, vaginal washes were collected from immunized and infected mice 2-days post-infection. From mice infected with the HSV-2_WT_, HSV-2_ΔmgG-2_ or HSV-2_rescue_ (see below) vaginal fluids were collected at 12 h and at 1-, 2-, 3-, 4-, 5- and 6-days after infection. Washes were obtained by gently pipetting 40 μl of Hanks balanced salt solution (HBSS) in and out of the vagina until a clump of mucus was retrieved, followed by a second wash with an additional 40 μl. Both washes were pooled and collected in a total volume of 1 ml HBSS. Samples were stored at − 80 °C until analysis. Also, the vaginas from mice infected with HSV-2_WT_ or HSV-2_ΔmgG-2_ were excised and homogenized (see section Tissue sample collection for HSV-2 DNA quantification below) and assessed for infectious viral particles using a plaque assay. To perform the plaque assay, samples were diluted 1:10 in PBS and added to 80% confluent monolayers of GMK-AH1 cells grown in 6- or 24-well plates, after which the plate was incubated at room temperature for 1 hour. Methylcellulose was mixed with EMEM (ThermoFisher Scientific) supplemented with 1% PEST and 2% FCS, and a total of 2 mL (6-well plates) or 400 µL (24-well plates) were added on top of the cultured cell layer. The plates were incubated for 72 h at 37 °C and 4% CO_2_ before removal of the methylcellulose overlay and staining with one drop of crystal violet per well. Thereafter the cells were washed with tap water four times and allowed to dry. The plaques were counted using a light microscope at four times magnification.

### HSV-2 DNA quantification in tissue samples

The DRG and the spinal cord were collected from mice immunized with EXCT4-mgG-2, EXCT4-mgG-2(−N − O), EXCT4-mgG-2(-N), EXCT4-mgG-2(-O), or EXCT4-mgG-2(-SA), either when the mice presented a disease score ≥ 3, or upon termination of the experiment at day 14. Samples were stored at -80 °C until further analysis. Ganglia and spinal cord were separately transferred to MagNa Lyser Green Bead tubes (Roche) and mixed with lysis buffer from the MagNa Pure LC DNA Isolation Kit II Tissue (Roche). The tissues were homogenized twice for 54 s at 6500 rpm in a MagNa Lyser (Roche). Viral DNA was extracted on a MagNa Pure LC Robot (Roche) together with the MagNA Pure LC DNA Isolation Kit I for DNA from serum or vaginal washes, or with the MagNA Pure LC DNA Isolation Kit II Tissue for DNA from tissue samples, according to the instructions from the manufacturer. Isolated DNA was stored at -80 °C until further analysis.

Quantification of viral DNA was performed with the 7300 Real-Time PCR System (Applied Biosystems) in a 50 µL reaction that contained 10 µg extracted DNA Universal Mastermix (ThermoFisher), and a TaqMan probe and primers with the following sequences; forward primer, 5´-TGC AGT TTA CGT ATA ACC ACA TAC AGC; reverse primer, 5´-AGC TTG CGG GCC TCG TT; and the probe, 5´-CGC CCC AGC ATG TCG TTC ACG-VIC-TAMRA [73]. For standardization of HSV-2 DNA to cell content a beta-globin gene system was constructed from a conserved region of the gene. The primers were as follows: forward; 5´-CTG AAA CAC TAT GGT GGA GCT CA, and reverse, 5´-AAC ACC AAG TTC TTC TGC CTT CAC. The probe, sequence 5´-TGC AGA GGA GAA GGC AGC CAT CAC T-FAM-BHQ1 (black hole quencher 1). The PCR reaction was run for 2 minutes at 50 °C, 10 minutes at 95 °C, followed by 45 cycles of 15 seconds at 95 °C and 1 minute at 58 °C.

In addition, Plasmids (pUC57) containing the target sequences were constructed (GenScript) and amplified in E. coli XL-1 Blue, purified by HiSpeed Plasmid Maxi Kit 7 (Qiagen) and quantified by spectrophotometer analysis. A standard curve based on six five-fold dilutions of the plasmid using an initial concentration of 1 x 10^6^ HSV-2 DNA and beta-globin copy numbers per reaction was included in each run. For tissue samples from lymph nodes and spinal cord we observed an inhibition of the PCR reaction probably due to high content of cellular DNA. These samples were therefore diluted 1:4 in PBS before extraction of HSV-2 DNA.

### Mouse model to assess T-cell specific immune responses

The vaccine candidates EXCT4-mgG-2 and EXCT4-mgG2(−N − O) were tested in 8-week-old female C57BL/6 mice (Charles River, Wilmington, USA). Animals were kept at the Astrid Fagraeus laboratory, Karolinska Institutet, in accordance with the recommendations of the Swedish Board of Agriculture. The protocol was approved by the local ethics committee, and all animal procedures were performed according to the approved guidelines and regulations. Protein antigen preparations were formulated in CpG1826 and alum (both from Invivogen) and inoculated intramuscularly in the gastrocnemius muscle of the left hind leg, three times at 10-day intervals at the doses of 2.5 μg antigen per mouse.

### Isolation of mouse splenocytes

Mouse spleens were harvested 14-days post the last immunization and mashed through 70-μm-pore-size nylon cell strainers to obtain single-cell suspensions. Cells were washed in complete RPMI medium (RPMI 1640 supplemented with 5% FCS, 2 mM l-glutamine, 100 U/ml penicillin, and 100 μg/ml streptomycin (all from Gibco/Invitrogen), followed by treatment with red blood cell lysis buffer (Sigma Aldrich) for 2 min. After another wash, the cells were resuspended in complete RPMI medium. Viable cells were quantified by using Guava Viacount and Guava cell counter (Merck-Millipore).

### FluoroSpot analysis

Mouse IFN-γ/IL-2/TNF FluoroSpot assay was performed using a commercial kit (Mabtech) according to the manufacturer’s instructions. Briefly, pre-coated plates for IFN-γ, IL-2, and TNF were washed five times with PBS. Splenocytes were plated at the concentration of 200 000 cells per well and stimulated with EXCT4-mgG-2 antigen 4µg/ml, EXCT4 peptide pool 4µg/ml (GenScript), ConA 4µg/ml (Merck-Millipore) or complete RPMI medium, then incubated for approximately 42 h at 37 °C in 5% CO₂. After incubation, cells were removed, and plates were washed before the addition of fluorophore-conjugated detection antibodies. Following further washing and drying, spots were visualized and quantified using a FluoroSpot reader (Mabtech IRIS).

### Intracellular cytokine assay

Freshly isolated splenocytes were set up at 2 × 10^6^ splenocytes per well in complete RPMI medium in 96-well round-bottom plate. Samples were incubated overnight at 37 °C in 5% CO₂ with 2 µg/ml EXCT4-mgG-2 peptide pool or complete medium alone. Cells were incubated with Protein Transport Inhibitor Cocktail (eBiosciences) for 4 hours. Cells were washed with FACS buffer and incubated with anti-CD3e-PerCP-Cy5.5 (145-2C11), anti-CD4-FITC (GK1.5), and anti-CD8-APC (53-6.7) in FACS buffer (all monoclonal antibodies were purchased from eBiosciences). After the excess antibody was washed away, the cells were fixed with Intracellular Fixaton & Permeabilization Buffer (eBiosciences) according to the manufacturer’s instructions. Samples were then stained with anti-IFN-γ-PE (XMG1.2) (eBiosciences). After being washed the cells were resuspended in FACS buffer and analysed with the Guava cell counter (Merck-Millipore).

### Frame shift mgG-2 negative HSV-2 mutant and rescue

An mgG-2 negative mutant was selected from the wild type HSV-2 strain 333 (referred in this study as HSV-2_WT_ (Fig 1A)) in GMK-AH1 cells in the presence of a sulphated oligosaccharide. This mutant, designated as AC9 (accession number EU018128), presented a deletion of a single nucleotide C (frame shift mutation) within a run of seven cytosine nucleotides (nts) at 1649–1655 resulting in a premature termination codon (TAA) at nts 1924–1926. A PCR amplified fragment encompassing nts 222–2140 of the gG-2 gene from strain AC9 was introduced back to HSV-2_WT_ by a marker transfer assay [34]. This virus is designated in this study as HSV-2_ΔmgG-2_ (Fig 1B). To rescue the HSV-2_ΔmgG-2_ mutant the gG-2 gene fragment (nts 222–2140) from the HSV-2_WT_ genome was amplified by PCR and co-transfected together with HSV-2_ΔmgG-2_ genomic DNA into GMK-AH1 cells. The selection of positive variants was based on a plaque assay using an anti-mgG-2 MAb (O1.C5.B2) [28] followed by the amplification of the gG-2 gene and DNA sequencing to confirm the wild type sequence. The marker rescued HSV-2 is designated in this study as HSV-2_rescue_. Primers for sequencing of the gG-2 gene were used as described [40].

As shown in the schematic illustration of the gG-2 proteins for strain HSV-2_ΔmgG-2_ (Fig 1B) the mutation disrupts the protein sequence of mgG-2 from amino acid 552 including the immunodominant region (amino acids 551–573), the transmembrane region as well as the intracellular region. The HSV-2_ΔmgG-2_ showed to lack expression of mgG-2 [34, 42]. In addition, as the viral gG and Us3 genes are expressed from the same bicistronic gene the normal expression of the Us3 protein was confirmed earlier [42]. To investigate that sgG-2 is not affected by the frame shift mutation in mgG-2 we infected GMK-AH1 cells at a MOI of 1 using HSV-2_ΔmgG-2_, HSV-2_rescue_ and HSV-2_WT_. After 24–36 h, at complete cytopathic effect, cell culture medium was centrifuged at 4000 x g. As the amount of PFU at complete cytopathic effect is different for the three strains we normalized the amount of supernatant to equal PFU with the ratio 1.0 for HSV-2_ΔmgG-2_ (titre 3.4 x 10^6^), ratio 0.17 for the HSV-2_WT_ (titre 2.0 x 10^7^), and ratio 0.074 for the HSV-2_rescue_ strain (titre 4.6 x 10^7^). The supernatant was mixed with sample buffer containing SDS and mercaptoethanol, followed by boiling 10 min and separated on a 4–12% NuPAGE Bis-Tris gradient gel (Invitrogen) with 3-(N-morpholino) propane sulfonic acid and SDS as running buffer. The proteins were transferred to an immobilon-PVDF membrane. A pre-stained protein ladder (ThermoFisher Scientific) was used as molecular weight marker. The anti-sgG-2 MAb (4.A5.A9) [27] was used for detection, at a 1:2000 dilution, and HRP-conjugated polyclonal anti-mouse IgG was used as conjugate (DakoCytomation), at a 1:20 000 dilution. After washing, SuperSignal West Dura Extended Duration Substrate (ThermoFisher Scientific) was added followed by detection using the ChemiDoc MP Imaging System (BioRad Laboratories).

### Mouse model of genital HSV-2 infection

In this study we used both C57BL/6 and DBA/2 mice. In an initial study, Lopez showed that after intraperitoneal inoculation of HSV-1 of different inbred mouse strains C57BL/6 was far more resistant to neuroinvasion and death as compared to the DBA mice [74]. The resistance in the C57BL/6 mice was later on found to be dependent on an early interferon response [75].

Female 6- to 8-week-old C57BL/6 mice (Scanbur BK) were used. The animals were kept at Experimental Biomedicine, Gothenburg University, the protocol was approved by the local ethics committee, and all procedures were performed according to approved guidelines and regulations. The mice were anesthetized with 3% isoflurane (Baxter). Before infection 3 mg of Depo-Provera was given subcutaneously, and 6 days later 4 x 10^4^ PFU (corresponding to 10 x lethal dose 50% (LD_50_), as determined for HSV-2_WT_) of HSV-2_WT_, HSV-2_ΔmgG-2_ or HSV-2_rescue_ were given intravaginally with a blunted fine plastic pipette. In addition, ten C57BL/6 mice were infected with a higher dose of 10 × 10^4^ PFU of HSV-2_ΔmgG-2_. We also used the more sensitive mouse strain DBA/2 (Harlan Lab) and infected 6- to 8-weeks old female mice intravaginally with 1.25 x 10^4^ PFU (corresponding to 125 x LD_50_, as determined for HSV-2_WT_) of HSV-2_WT_, HSV-2_ΔmgG-2_ or HSV-2_rescue_. The natural course of the vaginal HSV-2 infection is described in detail by Parr and Parr [39]. Briefly, HSV-2 infects initially the vaginal epithelium followed by spread to vulva, anus, and perineum, and to the autonomic and to the sensory neurons in DRG and the spinal cord. Mice were scored as described above.

### Tissue sample collection for HSV-2 DNA quantification

For the C57BL/6 mice, the vagina was excised at 12 h and at 1-, 2-, 3-, 4-, 5- and 6-days after infection and kept in 1 mL PBS at -80 °C. DRG and spinal cord were collected at 3- or 6-days after infection with HSV-2_WT_ or HSV-2_Δmg-G_ and at 14- or 21-days after infection of C57BL/6 mice infected with HSV-2_Δmg-G_. From C57BL/6 mice, serum was drawn daily from day 1 to day 6 and inguinal lymph nodes were collected at day 1 or day 6. From DBA/2 mice serum and inguinal lymph nodes were collected at day 6 post infection. All tissue samples were kept at -80 °C, thawed and homogenized by using a Dounce homogenizer prior to DNA extraction using the MagnaPure LC Robot (Roche) and DNA quantification using qPCR as described above.

### Tissue preparation, histology, and immunohistochemistry

DBA/2 mice were infected intravaginally with 1.25 x10^4^ PFU of HSV-2_ΔmgG-2_ or HSV-2_rescue_. At day 6 the animals were deeply anesthetized with isoflurane and fixed by transcardial perfusion via the left ventricle with PBS, followed by 4% formaldehyde. The spinal cord including DRG was removed, and immersion fixed for about 4 days. After fixation the tissue was decalcified, using 10% EDTA in 0.2 M Tris buffer, pH 7.4 for 3–4 weeks; the buffer was changed twice weekly. After decalcification, 3–4 mm thick horizontal slices were cut from the lumbosacral, the lumbar and thoracic level of the spinal cord. The specimens were dehydrated, infiltrated, and embedded in paraffin using a Leica TP1020 Automatic Tissue Processer (Leica Biosystems). Sections were cut at 4 μm and, after high temperature antigen retrieval, using a 0.01 M citrate buffer, pH 6.0, sections were incubated with rabbit polyclonal antibodies against HSV-2 antigen diluted 1/4000 (DakoCytomation). Anti-rabbit Impress Reagent HRP (Vector laboratories) was used as secondary reagents, and the immunoreactions were visualized using liquid DAB+ substrate (DakoCytomation). Nuclei were counter-stained with haematoxylin. Finally, the sections were dehydrated and mounted using DPX (Merck). A Zeiss Axio Image M1 microscope and AxioVision software (Zeiss) was used for documentation.

### Statistics

The SPSS 16 (IBM) and the GraphPad Prism 10.4.1 (Dotmatics) software programs were used for statistical calculations. Mann-Whitney or the Kruskal-Wallis (non-parametric) tests were used for comparison of HSV-2 viral load (PFU and HSV-2 DNA genome copies), and for comparison of IgG levels. The area under curve method based on the trapezoidal rule included in SigmaPlot or GraphPad Prism 11.0.0 was used for calculation of the ratio of PFU in vagina and vaginal washes, with statistical analysis performed using the Mann-Whitney test. For survival, the pairwise Log-rank (Mantel-Cox) test was used to test significantly altered survival as compared to mice immunized with EXCT4-mgG-2 or infected with HSV-2_WT_. For the fluorospot and intracellular cytokine assays, the data passed as normally distributed using the Shapiro-Wilk test, and significance was tested using the Tukey`s multiple comparison test. P-values < 0.05 were considered statistically significant.

## Supporting information

S1 Table Observed O-glycopeptides in the EXCT4-mgG-2 preparation. CV is calculated based on the percentual distribution within each injection (n = 3). For peptides with multiple possible glycan sites, one glycan structure was identified but the exact position cannot be determined.
(PDF)

S2 Table Observed N-glycopeptides in the EXCT4-mgG-2 preparation. CV is calculated based on the percentual distribution within each injection (n = 3).
(PDF)

S1 Fig Representative MS2 spectra of EXCT4-mgG-2 glycopeptides. (A) Fragmentation analysis of [L].GPLAPNTPRPPA.[Q] containing a di-sialylated core 1 O-glycan at T615. (B) Fragmentation analysis of [A].AAATPGAGHTNTS.[S] containing a mono-sialylated and core-fucosylated complex biantennary N-glycan at N436. The precursor ion structures are shown in the boxes. Ion charges are indicated when z > 1.
(PDF)

S2 Fig Verification of the enzymatic removal of glycan structures. (A) Western blot showing size shift of the N- and O-glycosylated EXCT4-mgG-2 and of the deglycosylated EXCT4-mgG-2(-N), EXCT4-mgG-2(-O), EXCT4-mgG-2(-SA), and EXCT4-mgG-2(−N − O). (B) Lectin blot confirming the removal of specific glycan structures. ConA; Binds to core oligomannose of N-linked glycans, Jacalin; binds to Tn and sialylated Tn-antigens of O-linked glycans, MAL II; binds to sialic acids.
(PDF)

S3 Fig Immunization with EXCT4-mgG-2 decorated with N-linked or O-linked glycans, or glycan devoid of sialic acids confer protection against viral challenge. C57BL/6 mice were intramuscularly immunized with EXCT4-mgG-2(-N), EXCT4-mgG-2(-O) and EXCT4-mgG-2(SA) and genitally challenged with 25 x LD_50_ of HSV-2_WT_. The survival rate (A) and disease score (B) was assessed until 15 d.p.i. Viral spread to neuronal tissue; HSV-2 DNA copies per ganglia (C) HSV-2 DNA copies per spinal cord (D). Statistical analysis was performed with the pairwise log-rank (Mantel Cox) (A) or Kruskal-Wallis test (C-D). The detection limit for HSV-2 DNA in ganglia and spinal cord was 40 and 160 copies respectively. D.p.i = Days past infection. Values are expressed as means ± SEM.
(PDF)

S4 Fig Representative flow cytometric analysis of intracellular INF-γ stained CD4 + T cells. Outlined is the gating strategy used for both CD4+ and CD8 + T cells.
(PDF)

S5 Fig Serum antibody reactivity against the glycosiadases present in the EXCT4-mgG-2(−N − O) preparation. Serum samples was collected 14 days following immunization with a preparation containing glycosidases, but no recombinant mgG-2, and the reactivity against the EXCT4-mgG-2 and the EXCT4-mgG-2(−N − O) assessed. Presented is mean ± SEM.
(PDF)

S6 Fig Western blot of cell lysates and growth medium after infection of Hep-2 cells with HSV-2_WT_, HSV-2_𝛥__mgG-2_ or HSV-2_rescue_. Anti-sgG-2 mAb recognized sgG-2 (44 kDa). One experiment of two performed is shown.
(PDF)

S3 Table Summary of the infectious doses of HSV-2_WT_, HSV-2_𝛥__mgG-2_ or HSV-2_rescue_ along with survival, disease score and PFU in vaginal washings for C57BL/6 and DBA mice. (PDF)

S4 Table Determination of infectious viral particles in neuronal tissue (spinal cord and ganglia) from C57BL/6 mice infected with HSV-2_𝛥__mgG-2_ at day 6, 14, and 21 post infection. (PDF)

S7 Fig Unprocessed original gels corresponding to S1 and S6 Figs. The images are presented in full without cropping or any additional image processing.
(PDF)
