# Correction: Glycoprotein G enables HSV-2 neuroinvasion and provides protection as a glycosylated vaccine antigen

**DOI:** 10.1371/journal.ppat.1014765

**Published:** 2026-11-05

**Authors:** Ebba Könighofer, Carolina Gustafsson, Lindvi Gudmundsdotter, Ekaterina Mirgorodskaya, Jonas Nilsson, Maria Ekblad, Beata Adamiak, Eva Jennische, Stefan Lange, Edward Trybala, Staffan Görander, Tomas Bergström, Jan-Åke Liljeqvist, Rickard Nordén

Older versions of Figs 3 and 5 were inadvertently published. The figure legend remain correct. The authors have provided a corrected version of the figures here.

**Fig 3:**
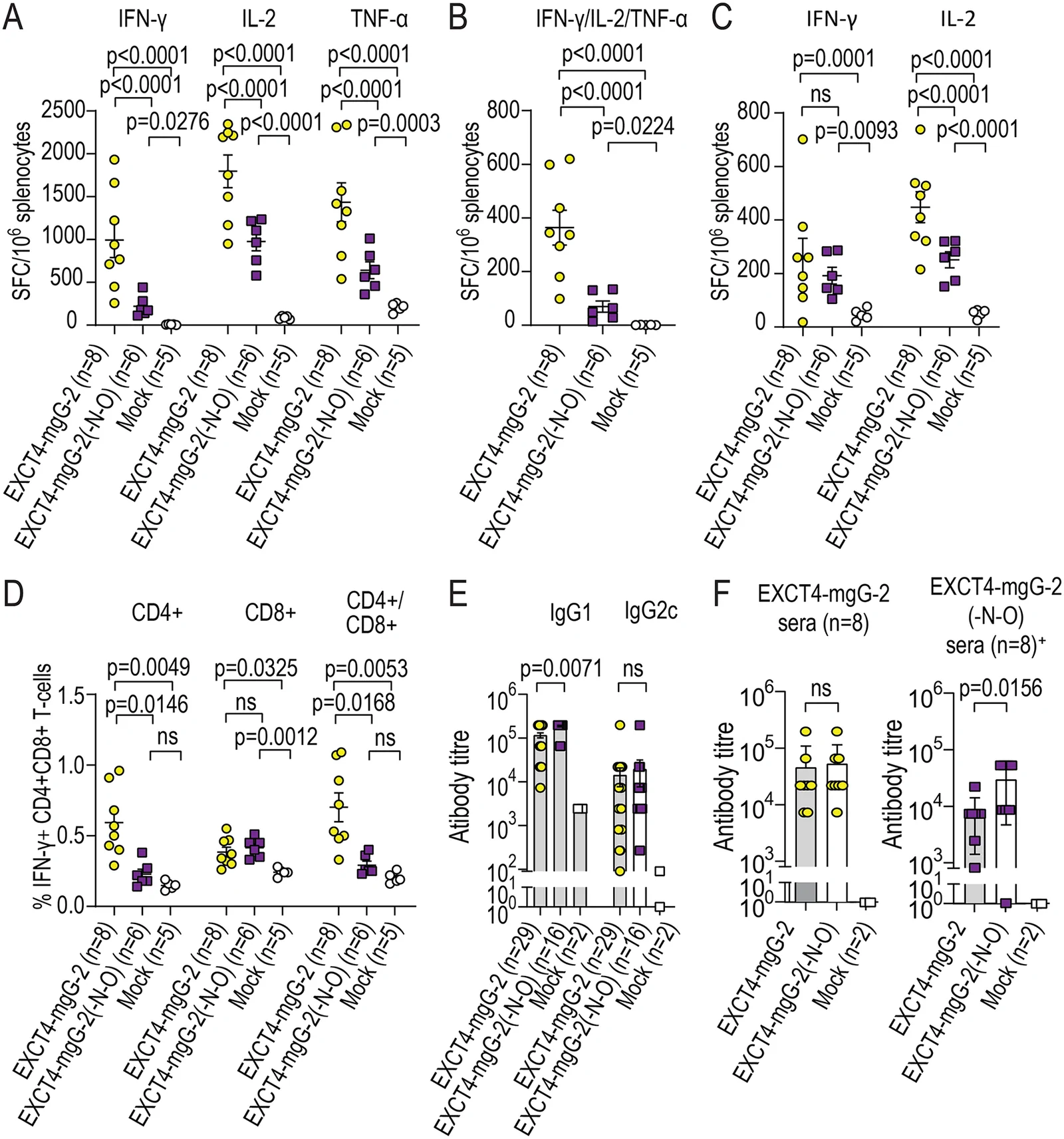
Immunization with EXCT4-mgG-2 induces Th1 polarization, a response which is attenuated in the absence of glycosylation. (A) Flourospot analysis of splenocytes harvested after immunization and stimulated with a pool of overlapping peptides (15-mers) covering the entire EXCT4-mgG-2 sequence, showing INF-γ, IL-2 and TNF-α production. (B) Fluorospot analysis showing the combined expression of INF-γ, IL-2 and TNF-α in splenocytes stimulated with the 15-mer peptide pool (C) Fluorospot analysis showing INF-γ and IL-2 expression in splenocytes stimulated with the N- and O-glycosylated EXCT4-mgG-2 recombinant protein (D) Percentage of INF-γ positive CD4+ and CD8  +  T cells from splenocytes stimulated with the 15-mer peptide pool. The splenocytes were harvested 14 days after the third immunization with EXCT4-mgG-2, EXCT4-mgG-2(−N  −  O) or mock and stimulated with the peptide pool overnight. (E) IgG1 and IgG2c levels in serum samples collected 14 days after the third intra muscularly (i. m.) immunization with EXCT4-mgG-2 or EXCT4-mgG-2(−N  −  O). (F) Serum antibody reactivity against EXCT4-mgG-2 and EXCT4-mgG-2(−N  −  O) was assessed in serum samples collected 14 days following immunization with EXCT4-mgG-2 or EXCT4-mgG-2(−N  −  O). + For immunization group EXCT4-mgG-2(−N  −  O), the antibody reactivity was corrected by subtracting values corresponding to glycosidase-specific binding in serum from mice immunized with the glycosidase enzymes in absence of EXCT4-mgG-2. SFC  =  Spot forming cells. Statistical analysis was performed with Saphiro-wilk test and Tukey’s multiple comparison test (A-D), Kruskal-Wallis test (E) or Mann-Whitney test (F). Values are expressed as means  ±  SEM.

**Fig 5:**
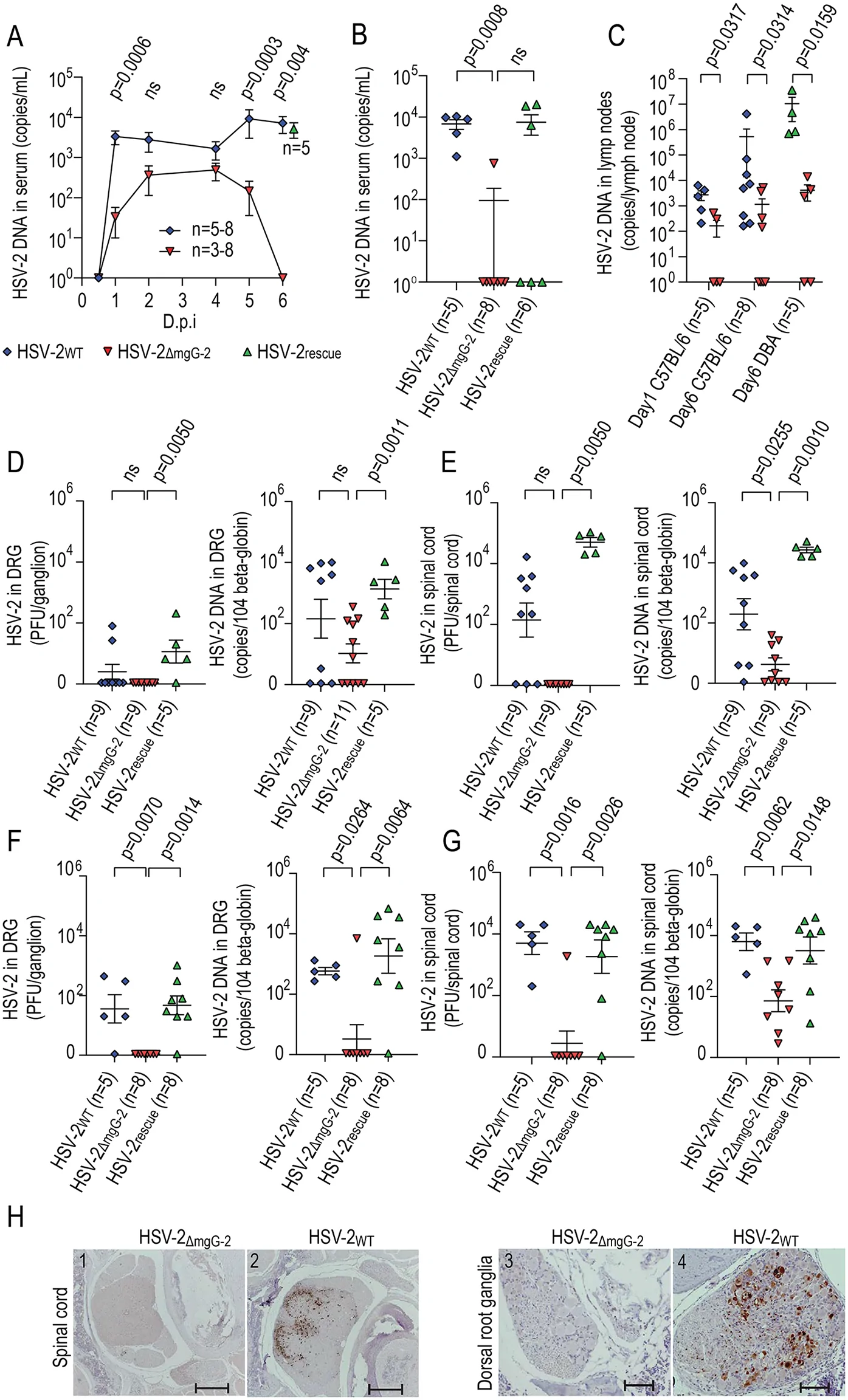
Assessment of the spread of HSV-2 to blood, genital lymph nodes, dorsal root ganglia and spinal cord, showing impaired capacity for neuronal spread of HSV-2_∆__mgG-2_. (A) C57BL/6 mice were infected with 10 x LD50 of HSV-2WT or HSV-2∆mgG-2, and HSV-2 DNA was calculated at 1–6 d.p.i. in serum. Sera from 5 C57BL/6 mice infected with the HSV-2rescue were analysed at day 6 (marked in green) (B) DBA/2 mice were infected with 125 x LD50 of HSV-2WT, HSV-2∆mgG-2 or HSV-2rescue, and HSV-2 DNA content in serum was calculated at 6 d.p.i. (C) C57BL/6 mice were infected with 10 x LD50 of HSV-2WT or HSV-2∆mgG-2 and HSV-2 DNA was calculated in genital lymph nodes at 1 or 6 d.p.i. (D-E) C57BL/6 mice were infected with 10 xLD50 and (F-G) DBA/2 mice were infected with 125 x LD50 of HSV-2WT, HSV-2∆mgG-2 or HSV-2rescue. Infectious HSV-2 and HSV-2 DNA copy numbers were analysed 6 d.p.i. (D and F) per single dorsal root ganglion and (E and G) in the whole spinal cord. (H) Paraffin sections from the vertebral column of DBA/2 mice 6 d.p.i. infection with (panel 1 and 3) HSV-2∆mgG-2 or (panel 2 and 4) with HSV-2rescue. HSV-2 antigens (brown) were visualized using a polyclonal rabbit anti-HSV-2 serum. Panel 1–2 show the spinal cord at the thoracic level and panel 3–4 show the dorsal root ganglia at the lumbosacral level from the same animal. After infection with HSV-2rescue several neurons and glial cells in the dorsal horns and neurons and satellite cells in the ganglia are infected. Bars: 1–2; 500 μm, 3–4; 100 μm. HSV-2 DNA copies are expressed per mL serum (detection limit 100 copies) or per lymph node (detection limit 80 copies). Values are expressed as means  ±  SEM. HSV-2 DNA copy numbers were standardized to 104 beta-globin genes. The virus detection limit was 40 PFU or 160 HSV-2 DNA copies per 3 ganglia or whole spinal cord. Statistical analysis is performed with Mann-Whitney test. Data are calculated from one to two independent experiments for each mouse strain except for the immunohistochemistry data where one experiment representative of the two performed is shown. D.p.i. - days post infection.
