# Supplementary material for: Glycoprotein G enables HSV-2 neuroinvasion and provides protection as a glycosylated vaccine antigen
Source: PLoS Pathog. 2026 Jul 9;22(7):e1014339. doi: 10.1371/journal.ppat.1014339 (PMC13349171; doi:10.1371/journal.ppat.1014339)
Supplement: S1 Table — CV is calculated based on the percentual distribution within each injection (n = 3). For peptides with multiple possible glycan sites, one glycan structure was identified but the exact position cannot be determined. (PDF) [file ppat.1014339.s001.pdf]

Table S1. Observed O-glycopeptides in the EXCT4-mgG-2 preparation. CV is calculated based on the percentual distribution within each injection (n = 3). For peptides with multiple possible glycan sites, one glycan structure was identified but the exact position cannot be determined.

Glycan site	Peptide Sequence	Glycan Composition	Theo. MH+ [Da]	Precursor ion Abundances (AU, Average for three injections)	Abundance (%, average for three injections)	Abundance CV (%)	Assigned glycan type
T356	[L].TEDASSDSPTSAPEKTPLPV.[S]	NG	2028,961	3,66E+06	5,72	6,71	
T356	[L].TEDASSDSPTSAPEKTPLPV.[S]	HexNAc(1)Hex(1)	2394,093	9,77E+06	15,27	10,38	O-linked
T356	[L].TEDASSDSPTSAPEKTPLPV.[S]	HexNAc(1)Hex(1) NeuAc(1)	2685,188	3,23E+07	50,63	5,03	O-linked
T356	[L].TEDASSDSPTSAPEKTPLPV.[S]	HexNAc(1)Hex(1) NeuAc(2)	2976,284	1,81E+07	28,38	3,55	O-linked
S411/T415	[A].VASPPATA.[S]	NG	713,3828	1,71E+06	2,41	23,71	
S411/T415	[A].VASPPATA.[S]	HexNAc(1)	916,4622	3,55E+06	4,97	9,66	O-linked
S411/T415	[A].VASPPATA.[S]	HexNAc(1)Hex(1)	1078,515	4,22E+06	5,90	7,55	O-linked
S411/T415	[A].VASPPATA.[S]	HexNAc(1)Hex(1) NeuAc(1)	1369,61	4,29E+07	59,47	1,74	O-linked
S411/T415	[A].VASPPATA.[S]	HexNAc(2)Hex(2)	1443,647	2,92E+06	4,07	6,44	2 x O-linked
S411/T415	[A].VASPPATA.[S]	HexNAc(1)Hex(1) NeuAc(1); HexNAc(1)Hex(1)	1734,743	9,05E+06	12,56	4,93	2 x O-linked
S411/T415	[A].VASPPATA.[S]	HexNAc(2)Hex(2) NeuAc(2)	2025,838	7,68E+06	10,63	3,59	2 x O-linked
S417/S420/S421	[A].SVESSPLPA.[S]	NG	886,4516	1,04E+07	22,50	6,60	
S417/S420/S421	[A].SVESSPLPA.[S]	HexNAc(1)	1089,531	2,80E+07	7,88	17,54	O-linked
S417/S420/S421	[A].SVESSPLPA.[S]	HexNAc(1)Hex(1)	1251,584	2,65E+07	1,12	16,57	O-linked
S417/S420/S421	[A].SVESSPLPA.[S]	HexNAc(1)Hex(1) NeuAc(1)	1542,679	9,09E+07	2,83	56,86	O-linked
S417/S420/S421	[A].SVESSPLPA.[S]	HexNAc(1)Hex(1) NeuAc(2)	1833,775	3,91E+06	13,26	2,44	O-linked
T445/T448/T449/T453/T454	[A].AKTPPTTPAPTTPPPTST.[H]	NG	1762,922	8,51E+06	86,66	0,73	
T445/T448/T449	[A].AKTPPTTPAPTTPPPTST.[H]	HexNac(1)Hex(1)	1966,002	1,31E+06	13,34	4,77	O-linked

/T453/T454							
T529/T532/T533	[R].TPPTDPKTHPHGPA.[D]	NG	1452,723	5,44E+08	31,03	5,39	
T529/T532/T533	[R].TPPTDPKTHPHGPA.[D]	HexNAc(1)	1655,802	2,21E+08	12,67	6,44	O-linked
T529/T532/T533	[R].TPPTDPKTHPHGPA.[D]	HexNAc(1)Hex(1)	1817,855	2,49E+08	14,16	5,42	O-linked
T529/T532/T533	[R].TPPTDPKTHPHGPA.[D]	HexNAc(2)	1858,882	7,50E+07	4,28	4,87	2 x O-linked
T529/T532/T533	[R].TPPTDPKTHPHGPA.[D]	HexNAc(2)Hex(1)	2020,935	2,00E+08	11,36	15,60	2 x O-linked
T529/T532/T533	[R].TPPTDPKTHPHGPA.[D]	HexNAc(1)Hex(1) NeuAc(1)	2108,951	3,63E+07	2,08	10,74	O-linked
T529/T532/T533	[R].TPPTDPKTHPHGPA.[D]	HexNAc(2)Hex(2)	2182,987	2,72E+08	15,52	10,42	2 x O-linked
T529/T532/T533	[R].TPPTDPKTHPHGPA.[D]	HexNAc(2)Hex(2) NeuAc(1)	2474,083	1,29E+08	7,33	11,51	2 x O-linked
T529/T532/T533	[R].TPPTDPKTHPHGPA.[D]	HexNAc(2)Hex(2) NeuAc(2)	2765,178	2,05E+07	1,16	14,95	2 x O-linked
S548	[A].DAPPGSPAPPPPEHR.[G]	NG	1521,744	3,26E+07	2,02	8,03	
S548	[A].DAPPGSPAPPPPEHR.[G]	HexNAc(1)	1724,824	8,73E+07	5,37	6,90	O-linked
S548	[A].DAPPGSPAPPPPEHR.[G]	HexNAc(1)Hex(1)	1886,877	4,19E+08	25,80	1,05	O-linked
S548	[A].DAPPGSPAPPPPEHR.[G]	HexNAc(1)Hex(1) NeuAc(1)	2177,972	9,58E+08	59,01	0,51	O-linked
S548	[A].DAPPGSPAPPPPEHR.[G]	HexNAc(1)Hex(1) NeuAc(2)	2469,068	1,27E+08	7,80	2,68	O-linked
T615	[L].GPLAPNTPRPPA.[Q]	NG	1187,653	2,81E+06	1,95	8,16	
T615	[L].GPLAPNTPRPPA.[Q]	HexNAc(1)	1390,733	6,30E+06	4,40	8,22	O-linked
T615	[L].GPLAPNTPRPPA.[Q]	HexNAc(1)Hex(1)	1552,785	4,38E+07	30,55	4,42	O-linked
T615	[L].GPLAPNTPRPPA.[Q]	HexNAc(1)Hex(1) NeuAc(1)	1843,881	6,46E+07	45,11	2,88	O-linked
T615	[L].GPLAPNTPRPPA.[Q]	HexNAc(1)Hex(1) NeuAc(2)	2134,976	2,58E+07	17,98	0,46	O-linked
S629/T632	[A].KDMPSGPTPQHIPL.[F]		1517,778	7,84E+06	1,32	2,09	O-linked
S629/T632	[A].KDMPSGPTPQHIPL.[F]	HexNAc(1)Hex(1)	1882,91	2,81E+07	4,72	2,13	O-linked
S629/T632	[A].KDMPSGPTPQHIPL.[F]	HexNAc(1)Hex(1) NeuAc(1); HexNAc(1)Hex(1) NeuAc(2)	3121,329	1,35E+07	2,28	5,37	O-linked
S629/T632	[A].KDMPSGPTPQHIPL.[F]	HexNAc(1)Hex(1) NeuAc(1)	2174,006	2,15E+08	36,21	1,48	O-linked
S629/T632	[A].KDMPSGPTPQHIPL.[F]	HexNAc(1)Hex(1) NeuAc(2)	2465,101	3,04E+08	51,13	0,77	O-linked
S629/T632	[A].KDMPSGPTPQHIPL.[F]	HexNAc(2)Hex(2) NeuAc(2)	2830,233	2,17E+07	3,65	2,78	2 x O-linked
S643/S645	[W].FLTASPALD.[V]	NG	934,488	3,19E+06	14,15	10,98	
S643/S645	[W].FLTASPALD.[V]	HexNAc(1)Hex(1) NeuAc(1)	1590,716	8,39E+06	27,95	6,71	O-linked
S643/S645	[W].FLTASPALD.[V]	HexNAc(1)Hex(1) NeuAc(2)	1881,811	1,09E+07	48,50	1,03	O-linked

NG = Non glycosylated
