# Supplementary material for: Glycoprotein G enables HSV-2 neuroinvasion and provides protection as a glycosylated vaccine antigen
Source: PLoS Pathog. 2026 Jul 9;22(7):e1014339. doi: 10.1371/journal.ppat.1014339 (PMC13349171; doi:10.1371/journal.ppat.1014339)
Supplement: S2 Table — CV is calculated based on the percentual distribution within each injection (n = 3). (PDF) [file ppat.1014339.s002.pdf]

Table S2. Observed N-glycopeptides in the EXCT4-mgG-2 preparation. CV is calculated based on the percentual distribution within each injection (n = 3).

Glycan site	Peptide Sequence	Glycan Composition	Theo. MH+ [Da]	Precursor ion Abundances (AU, Average for three injections)	Abundance (%, average for three injections)	Abundance CV (%)	Assigned glycan type	Abundance (%) of complex type structures
N436	[A].AAATPGAGHT NTS.[S]	NG	1155,54	3,78E+05	4,06	138,81 [a]		
N436	[A].AAATPGAGHT NTS.[S]	HexNAc(2)Hex(3) Fuc(1)	2193,91	3,25E+06	28,12	12,60	N-linked, paucimannose	
N436	[A].AAATPGAGHT NTS.[S]	HexNAc(2)Hex(3) Fuc(1); HexNAc(1)	2396,99	2,92E+05	2,56	5,80	N-linked, Complex [b]	
N436	[A].AAATPGAGHT NTS.[S]	HexNAc(2)Hex(3) Fuc(1); HexNAc(1)Hex(1)	2559,05	2,64E+05	2,31	12,65	N-linked, Complex [b]	
N436	[A].AAATPGAGHT NTS.[S]	HexNAc(2)Hex(4)	2209,91	1,11E+06	9,77	2,78	N-linked, Oligomannose	
N436	[A].AAATPGAGHT NTS.[S]	HexNAc(2)Hex(5)	2371,96	2,34E+06	20,81	8,50	N-linked, Oligomannose	
N436	[A].AAATPGAGHT NTS.[S]	HexNAc(4)Hex(3) Fuc(1)	2600,07	5,40E+05	4,73	10,50	N-linked, Complex	14,49
N436	[A].AAATPGAGHT NTS.[S]	HexNAc(4)Hex(4) Fuc(1)	2762,13	1,05E+06	9,15	10,38	N-linked, Complex	28,05
N436	[A].AAATPGAGHT NTS.[S]	HexNAc(4)Hex(5) Fuc(1)	2924,18	1,57E+06	13,64	9,46	N-linked, Complex	42,05
N436	[A].AAATPGAGHT NTS.[S]	HexNAc(4)Hex(5) Fuc(1)NeuAc(1)	3215,27	5,75E+05	4,87	30,60	N-linked, Complex	15,42
N511	[S].AANVSVA.[A]	HexNAc(2)Hex(5)	1847,76	6,67E+06	100	0	N-linked, Oligomannose	

[a] The high CV among this peptide originates from a different peak-integration between injections. However, manual inspection of the peaks could confirm similar elution profiles and intensities in all three injections.

[b] Manual inspection of the ion spectra suggests a monoanntennary N-linked complex-type glycan.
