# Supplementary material for: Glycoprotein G enables HSV-2 neuroinvasion and provides protection as a glycosylated vaccine antigen
Source: PLoS Pathog. 2026 Jul 9;22(7):e1014339. doi: 10.1371/journal.ppat.1014339 (PMC13349171; doi:10.1371/journal.ppat.1014339)
Supplement: S1 Fig — (A) Fragmentation analysis of [L].GPLAPNTPRPPA.[Q] containing a di-sialylated core 1 O-glycan at T615. (B) Fragmentation analysis of [A].AAATPGAGHTNTS.[S] containing a mono-sialylated and core-fucosylated complex biantennary N-glycan at N436. The precursor ion structures are shown in the boxes. Ion charges are indicated when z > 1. (PDF) [file ppat.1014339.s003.pdf]

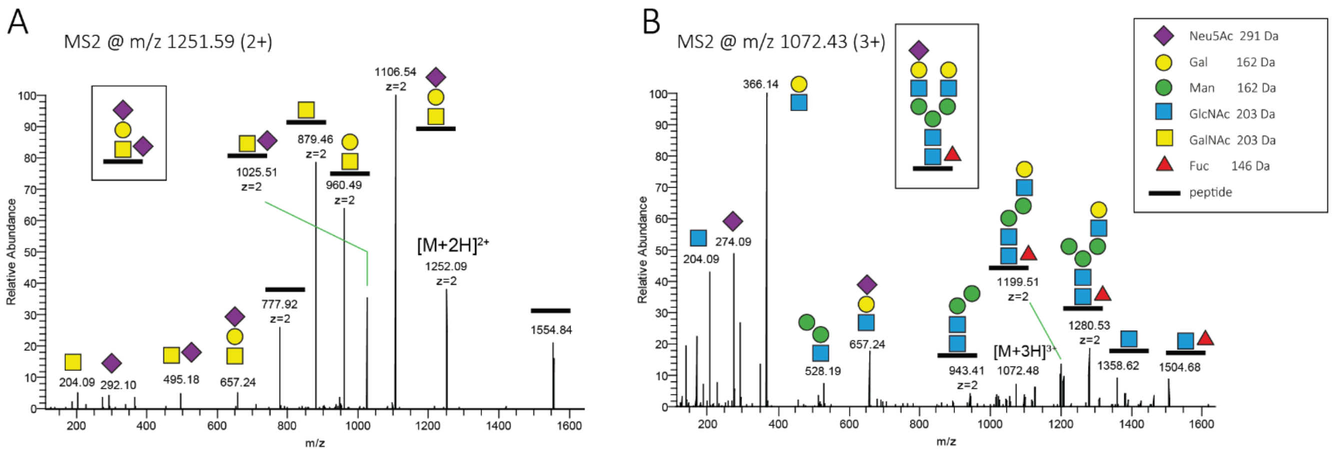

Figure S1. **Representative MS2 spectra of EXCT4-mgG-2 glycopeptides. (A)** Fragmentation analysis of [L].GPLAPNTPRPPA.[Q] containing a di-sialylated core 1 O-glycan at T615. **(B)** Fragmentation analysis of [A].AAATPGAGHTNTS.[S] containing a mono-sialylated and core-fucosylated complex biantennary N-glycan at N436. The precursor ion structures are shown in the boxes. Ion charges are indicated when z>1.
