# Supplementary material for: Glycoprotein G enables HSV-2 neuroinvasion and provides protection as a glycosylated vaccine antigen
Source: PLoS Pathog. 2026 Jul 9;22(7):e1014339. doi: 10.1371/journal.ppat.1014339 (PMC13349171; doi:10.1371/journal.ppat.1014339)
Supplement: S2 Fig — (A) Western blot showing size shift of the N- and O-glycosylated EXCT4-mgG-2 and of the deglycosylated EXCT4-mgG-2(-N), EXCT4-mgG-2(-O), EXCT4-mgG-2(-SA), and EXCT4-mgG-2(−N − O). (B) Lectin blot confirming the removal of specific glycan structures. ConA; Binds to core oligomannose of N-linked glycans, Jacalin; binds to Tn and sialylated Tn-antigens of O-linked glycans, MAL II; binds to sialic acids. (PDF) [file ppat.1014339.s004.pdf]

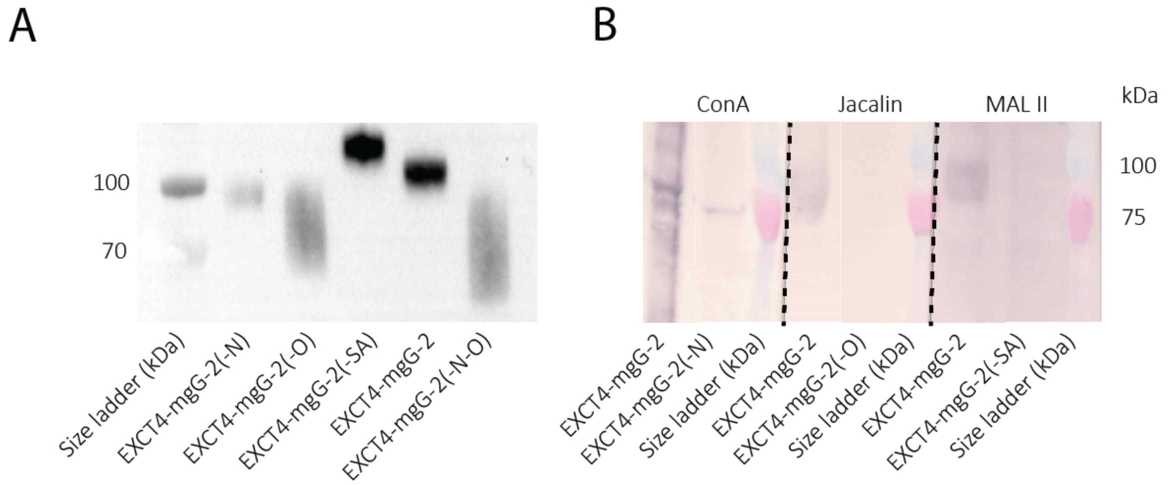

Figure S2. Verification of the enzymatic removal of glycan structures. (A) Western blot showing size shift of the N- and O-glycosylated EXCT4-mgG-2 and of the deglycosylated EXCT4-mgG-2(-N), EXCT4-mgG-2(-O), EXCT4-mgG-2(-SA), and EXCT4-mgG-2(−N−O). **(B)** Lectin blot confirming the removal of specific glycan structures. ConA; Binds to core oligomannose of N-linked glycans, Jacalin; binds to Tn and sialylated Tn-antigens of O-linked glycans, MAL II; binds to sialic acids.
