# Supplementary material for: Glycoprotein G enables HSV-2 neuroinvasion and provides protection as a glycosylated vaccine antigen
Source: PLoS Pathog. 2026 Jul 9;22(7):e1014339. doi: 10.1371/journal.ppat.1014339 (PMC13349171; doi:10.1371/journal.ppat.1014339)
Supplement: S3 Fig — C57BL/6 mice were intramuscularly immunized with EXCT4-mgG-2(-N), EXCT4-mgG-2(-O) and EXCT4-mgG-2(SA) and genitally challenged with 25 x LD50 of HSV-2WT. The survival rate (A) and disease score (B) was assessed until 15 d.p.i. Viral spread to neuronal tissue; HSV-2 DNA copies per ganglia (C) HSV-2 DNA copies per spinal cord (D). Statistical analysis was performed with the pairwise log-rank (Mantel Cox) (A) or Kruskal-Wallis test (C-D). The detection limit for HSV-2 DNA in ganglia and spinal cord was 40 and 160 copies respectively. D.p.i = Days past infection. Values are expressed as means ± SEM. (PDF) [file ppat.1014339.s005.pdf]

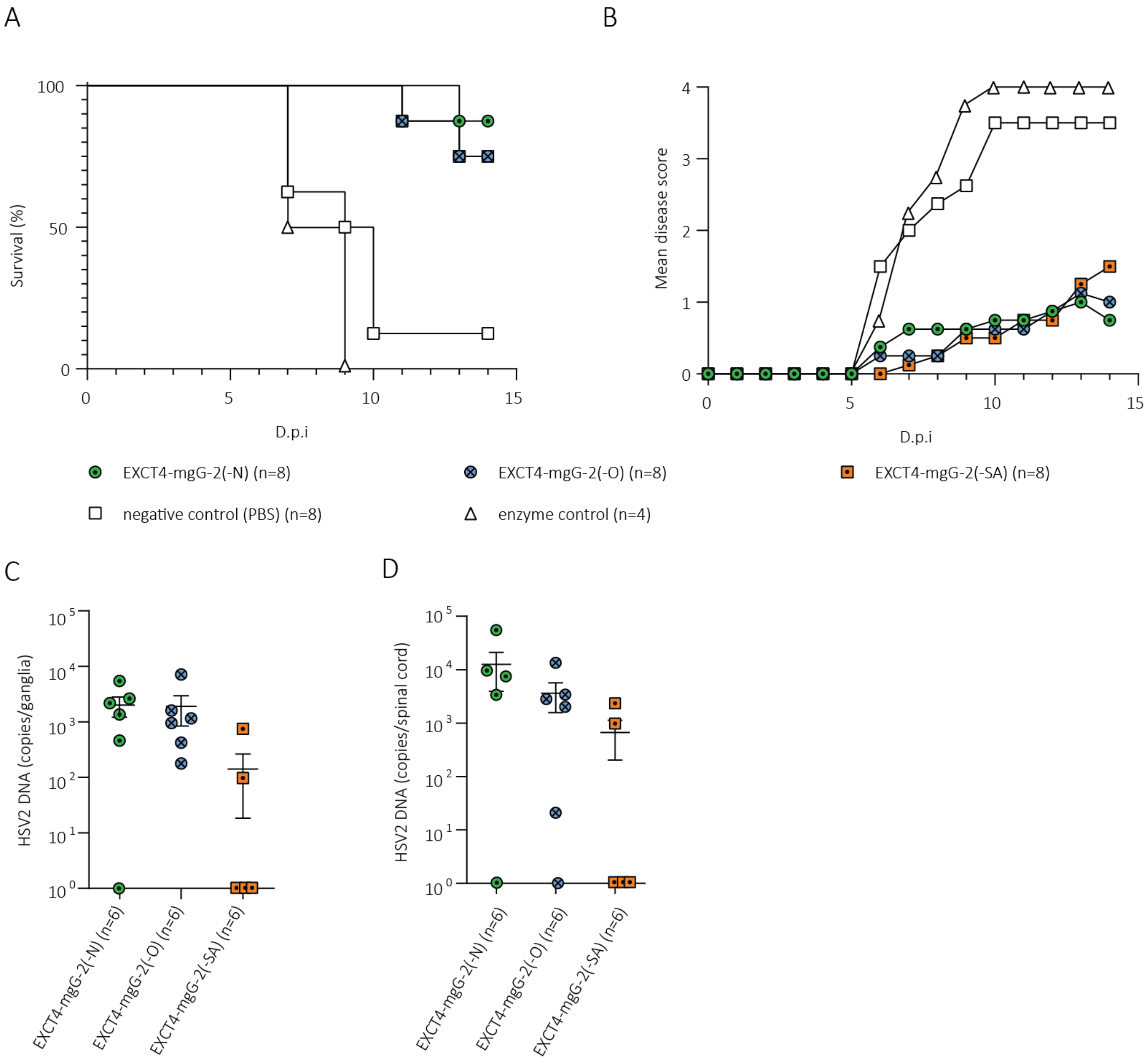

Figure S3. Immunization with EXCT4-mgG-2 decorated with N-linked or O-linked glycans, or glycan devoid of sialic acids confer protection against viral challenge. C57BL/6 mice were intramuscularly immunized with EXCT4-mgG-2(-N), EXCT4-mgG-2(-O) and EXCT4-mgG-2(SA) and genitally challenged with 25 x LD_{50} of HSV-2_{WT}. The survival rate **(A)** and disease score **(B)** was assessed until 15 d.p.i. Viral spread to neuronal tissue; HSV-2 DNA copies per ganglia **(C)** HSV-2 DNA copies per spinal cord **(D)**. Statistical analysis was performed with the pairwise log-rank (Mantel Cox) (A) or Kruskal-Wallis test (C-D). The detection limit for HSV-2 DNA in ganglia and spinal cord was 40 and 160 copies respectively. D.p.i = Days post infection. Values are expressed as means ± SEM.
