# Supplementary material for: Glycoprotein G enables HSV-2 neuroinvasion and provides protection as a glycosylated vaccine antigen
Source: PLoS Pathog. 2026 Jul 9;22(7):e1014339. doi: 10.1371/journal.ppat.1014339 (PMC13349171; doi:10.1371/journal.ppat.1014339)
Supplement: S4 Fig — Outlined is the gating strategy used for both CD4+ and CD8 + T cells. (PDF) [file ppat.1014339.s006.pdf]

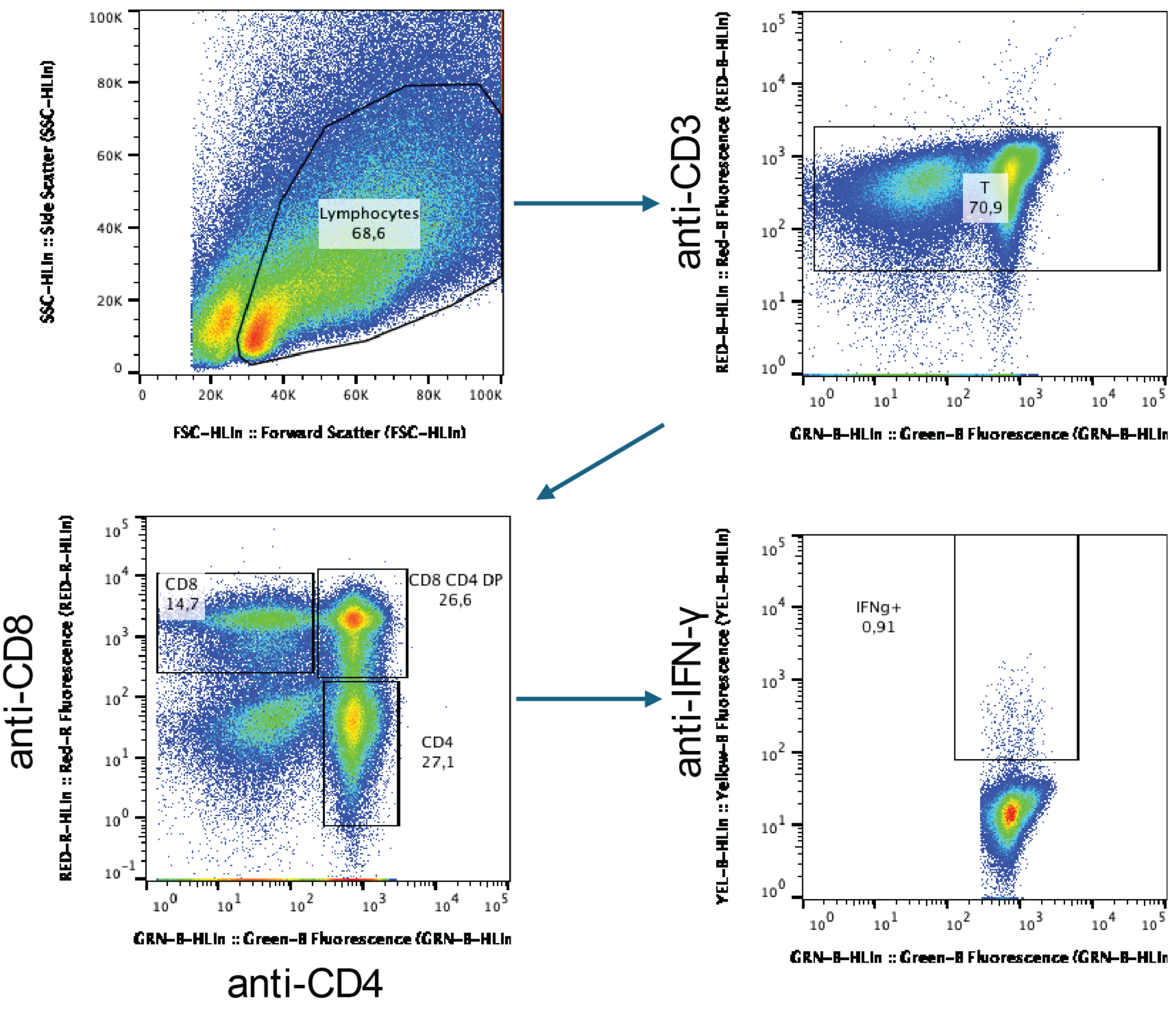

Figure S4. **Representative flow cytometric analysis of intracellular INF-γ stained CD4+ T cells.** Outlined is the gating strategy used for both CD4+ and CD8+ T cells.
