# Supplementary material for: Glycoprotein G enables HSV-2 neuroinvasion and provides protection as a glycosylated vaccine antigen
Source: PLoS Pathog. 2026 Jul 9;22(7):e1014339. doi: 10.1371/journal.ppat.1014339 (PMC13349171; doi:10.1371/journal.ppat.1014339)
Supplement: S5 Fig — Serum samples was collected 14 days following immunization with a preparation containing glycosidases, but no recombinant mgG-2, and the reactivity against the EXCT4-mgG-2 and the EXCT4-mgG-2(−N − O) assessed. Presented is mean ± SEM. (PDF) [file ppat.1014339.s007.pdf]

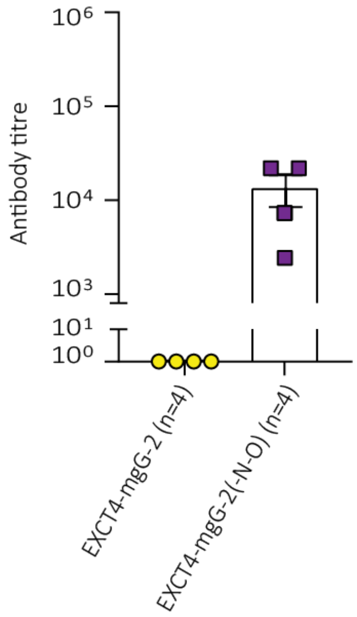

Figure S5. Serum antibody reactivity against the glycosidases present in the EXCT4-mgG-2(−N−O) preparation. Serum samples was collected 14 days following immunization with a preparation containing glycosidases, but no recombinant mgG-2, and the reactivity against the EXCT4-mgG-2 and the EXCT4-mgG-2(−N−O) assessed. Presented is mean ± SEM.
