# Supplementary material for: Glycoprotein G enables HSV-2 neuroinvasion and provides protection as a glycosylated vaccine antigen
Source: PLoS Pathog. 2026 Jul 9;22(7):e1014339. doi: 10.1371/journal.ppat.1014339 (PMC13349171; doi:10.1371/journal.ppat.1014339)
Supplement: S6 Fig — Anti-sgG-2 mAb recognized sgG-2 (44 kDa). One experiment of two performed is shown. (PDF) [file ppat.1014339.s008.pdf]

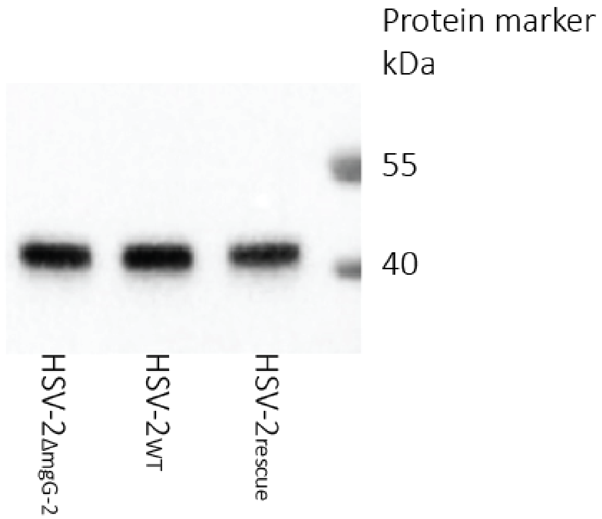

Figure S6. Western blot of cell lysates and growth medium after infection of Hep-2 cells with HSV-2$_{WT}$, HSV-2$_{\Delta mgG\text{-}2}$ or HSV-2$_{rescue}$. Anti-sgG-2 mAb recognized sgG-2 (44 kDa). One experiment of two performed is shown.
