# Supplementary material for: Glycoprotein G enables HSV-2 neuroinvasion and provides protection as a glycosylated vaccine antigen
Source: PLoS Pathog. 2026 Jul 9;22(7):e1014339. doi: 10.1371/journal.ppat.1014339 (PMC13349171; doi:10.1371/journal.ppat.1014339)
Supplement: S3 Table — (PDF) [file ppat.1014339.s009.pdf]

Table S3. Summary of the infectious doses of HSV-2$_{WT}$, HSV-2$_{\Delta mgG\text{-}2}$ or HSV-2$_{rescue}$ along with survival, disease score and PFU in vaginal washings for C57BL/6 and DBA mice.

Mice strain	Viral strain	Infectious dose	PFU/mL vaginal wash 2 d.p.i (n)	Survival 21 d.p.i% (n total)
C57BL/6	HSV-2$_{WT}$	40 000 PFU/mL	1163 (6)	0 (26)
	HSV-2$_{\Delta mgG\text{-}2}$	40 000 PFU/mL	43 (6)	100 (26)
		100 000 PFU/mL	1496 (10)	100 (10)
	HSV-2$_{rescue}$	40 000 PFU/mL	7504 (10)	0 (8)
DBA	HSV-2$_{WT}$	12 500 PFU/mL	5437 (7)	0 (7)
	HSV-2$_{\Delta mgG\text{-}2}$	12 500 PFU/mL	1524 (24)	86 (29)
	HSV-2$_{rescue}$	12 500 PFU/mL	5188 (16)	0 (22)

D.p.i = Days post infection.
