# Supplementary material for: Glycoprotein G enables HSV-2 neuroinvasion and provides protection as a glycosylated vaccine antigen
Source: PLoS Pathog. 2026 Jul 9;22(7):e1014339. doi: 10.1371/journal.ppat.1014339 (PMC13349171; doi:10.1371/journal.ppat.1014339)
Supplement: S4 Table — (PDF) [file ppat.1014339.s010.pdf]

Table S4. Determination of infectious viral particles in neuronal tissue (spinal cord and ganglia) from C57BL/6 mice infected with HSV-2$_{\Delta mgG-2}$ at day 6, 14, and 21 post infection.

Mice strain	Viral strain	Infectious dose	PFU/mL neuronal tissue 6 D.p.i (n)	PFU/mL neuronal tissue 14 D.p.i (n)	PFU/mL neuronal tissue 21 D.p.i (n)
C57BL/6	HSV-2$_{\Delta mgG-2}$	100 000 PFU/mL	0 (10)		
		40 000 PFU/mL		0 (5)	0 (5)

D.p.i = Days post infection.
