# Supplementary figures and images for: Glycoprotein G enables HSV-2 neuroinvasion and provides protection as a glycosylated vaccine antigen
Source: PLoS Pathog. 2026 Jul 9;22(7):e1014339. doi: 10.1371/journal.ppat.1014339 (PMC13349171; doi:10.1371/journal.ppat.1014339)

Original image supplemental Figure 1

A

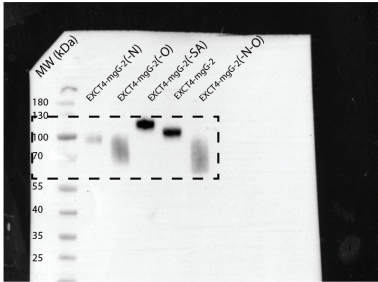

B

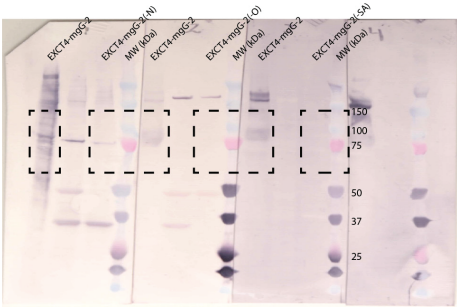

Original image supplemental Figure 6

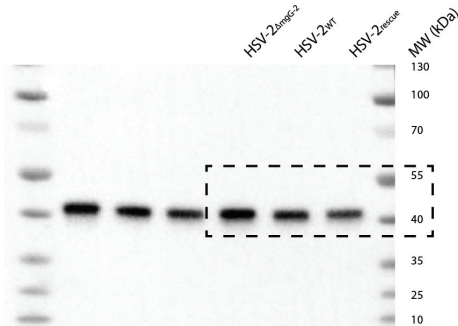

Supplement: S7 Fig — The images are presented in full without cropping or any additional image processing. (PDF) [file ppat.1014339.s011.pdf]
